# Supplementary material for: Impact of COVID-19 on health-related quality of life in the general population: A systematic review and meta-analysis
Source: PLOS Glob Public Health. 2023 Oct 26;3(10):e0002137. doi: 10.1371/journal.pgph.0002137 (PMC10602258; doi:10.1371/journal.pgph.0002137)
Supplement: S1 Table — (DOCX) [file pgph.0002137.s002.docx]

**S1 Table: Summary of included studies**

| **Citation** | **Country and Design** | **Sample size, female population** | **Age, population** | **Assessment tool** | **Statistical tests** | **Outcomes and risk factors** |
| --- | --- | --- | --- | --- | --- | --- |
| 1. Algahtani et al. 2020 | Saudi Arabia, Cross-sectional survey | 754, female 56% | General population | WHO QoL-BREF | Median and inter-quartile range were used to describe the QoL scores. A multinomial regression analysis was computed between QoL score quartiles and associated factors and the statistical significance was set at p < 0.05 | Males (OR = 1.96; 95% CI = 1.31–2.94); participants aged 26 to 35 years (OR = 5.1; 95% CI = 1.33–19.37); non-Saudi participants (OR = 1.69; 95% CI = 1.06–2.57); individuals with chronic diseases (OR = 2.15; 95% CI = 1.33–3.48); those who lost their job (OR = 2.18; 95% CI = 1.04–4.57); and those with depression (OR = 5.70; 95% CI = 3.59–9.05), anxiety (OR = 5.47; 95% CI = 3.38–8.84), and stress (OR = 6.55; 95% CI = 4.01–10.70) |
| 2. Abdullah et al. 2021 | Malaysia, Cross sectional | 326, ¾ (75%) females | Urban population | WHOQoL-BREF | Simple logistic regression and reported odds ratios as well as their confidence intervals | Higher psychological QoL reduced with the odds of depressive symptoms (adjusted OR = 0.83, 95% CI = 0.69–0.99, p = 0.032) and depressive with comorbid anxiety symptoms (adjusted OR = 0.82, 95% CI = 0.68–0.98, p = 0.041), whereas higher physical health QoL (adjusted OR = 0.85, 95% CI = 0.75–0.97, p = 0.021) and social relationship QoL (adjusted OR = 0.70, 95% CI = 0.55–0.90, p = 0.009) reduced the odds of anxiety symptoms |
| 3. Al-Shannaq et al 2020 | Jordan, cross sectional | 511, 65.2% female | Jordan general population | WHOQOL-BREF | Independent-samples analyzed using t-test for two groups and one-way ANOVA test for three or more groups.  Adjusted P-value for pairwise multiple comparisons was performed using the Bonferroni.  Multiple regression was performed to determine the significant predictors of depression, coping skills, and total QOL scores among the study participants | A mean for total QoL score of 73.21 (SD ¼ 16.17). The mean general QoL and health scores were 3.15 (SD ¼ 0.94) and 3.40 (SD ¼ 0.95).  40.7% of the participants described their QOL during the outbreak of COVID-19 as being neither poor nor good.  47.6% (n ¼ 243) of the participants reported feeling satisfied with their health.  mean scores in each domain of QoL were as follows: 18.04 (SD ¼ 4.39) for physical health, 17.65 (SD ¼ 3.77) for psychological health, 8.69 (SD ¼ 2.67) for social relationships, and 22.29 (SD ¼ 5.84) for environment |
| 4. Aruta et al. 2021 | Philippines, Cross sectional | 401, 63.34% female | Philipines, 18-68 age | MLT(My life Today) | CFA was conducted for each scale using maximum likelihood (MLT) estimation in Mplus 7.0  The goodness of data‐model fit was evaluated using CFI and TLI values between 0.90 and 0.95 as well as RMSEA and SRMR values of < 0.08 as criteria for an adequate model fit. CFI and TLI values > 0.95 and RMSEA and SRMR values < 0.05 were criteria for a good model fit  Reliability, and McDonald's omega (ω) were estimated to assess the internal consistency (Cronbach's α) of scores, with α and ω ≥ 0.70 indicating strong reliability  Bivariate correlation using Pearson r  latent variable path analysis (LVPA) through structural equation modeling (SEM) was conducted | Results of the path analysis indicated a good data‐model fit: (χ 2 = 4.97, df = 2, p = 0.08; CFI = 0.99, TLI = 0.96, SRMR = 0.02, RMSEA [90% CI] = 0.06 [0.000 − 0.13]).  The direct effects of safety at home (B = −0.27, β = −0.21, SE = 0.05, p ≤ 0.001), TPIs (B = −0.19, β = −0.27, SE = 0.05, p ≤ 0.001), and financial difficulties (B = 0.15, β = 0.18, SE = 0.05, p ≤ 0.001) on psychological distress were found to be significant  Direct effects of safety at home (B = 0.19, β = 0.22, SE = 0.05, p ≤ 0.001), TPIs (B = 0.18, β = 0.27, SE = 0.04, p ≤ 0.001), financial difficulties (B = −0.15, β = −0.21, SE = 0.05, p ≤ 0.001), and psychological distress (B = −0.29, β = −0.34, SE = 0.04, p ≤ 0.001) on quality of life were found to be significant  Results indicated that psychological distress partially mediated the positive influence of safety at home (B = 0.06, β = 0.07, SE = 0.02, p ≤ 0.001) and TPIs (B = 0.06, β = 0.09, SE = 0.02, p ≤ 0.001) on quality of life. |
| 5. Azizi et al 2020 | Morocco, Cross sectional | Before confinement( 484) and confinement ( 537)  62.9% female | Morocco population | EQ-5D-5L | For utility and VAS were tested using Mann-Whitney and Kruskal-Wallis tests were used | Before confinement, the percentages of don’t have problems in the five health dimensions were 87% (87%), 97% (93%), 82% (89%), 70% (78%) and 44% (66%) for mobility, self-care, usual activities, pain/discomfort and anxiety/depression, respectively  The comparison between the two samples showed that participants during confinement had lower scores of HRQoL on both utility (0.86; P<0.001)  However, the home confinement increased by about ARI = 7.1% [2.7%; 11%] the problems in doing usual activities (odds Ordinal = 1.74 [1.37; 2.23]).  Females had lower scores of HRQoL on both utility (0.85; P=<0.0001 and VAS (78.49;p=0.004) compared to males (utility =0.89 and VAS =83.78)  Marital status was significantly associated to EQ-5D-5L utility (P=0.002) and VAS (P=0.005) scores,  widowed had the worst HRQoL (utility=0.43 and VAS=48.75) compared to single (utility=0.87 and VAS=80.09), married (utility=0.86 and VAS=81.43), and separated (utility=0.89 and VAS=80.15) participants  Participants with university education had the higher EQ-5D-5L utility score (0.88; P<0.00001) |
| 6.Ballegooijen et al 2021 | Belgium and Netherlands , cross sectional | 2099 Belgian and 2058 Dutch respondents | Belgians and Dutch population, 50% were female | EQ-5D | Proportions and means with 95% CI, and means with SD  Analyses were performed separately for Belgium and the Netherlands  Subgroups of respondents were created based on age 18–35 years, 35– 66 years (for Belgium) and 35–67 (for the Netherlands), and above pension age ≥ 66/≥67 years, respectively | A minority in both countries felt stressed (27% in Belgium and 14% in the Netherlands)  Majority reported concern about their personal current and future financial situation (59 and 48% respectively)  and the national economies (88 and 86%)  Belgium  EQ-5D during COVID-19 measures 0.79 (0.77–0.81)  EQ-5D before COVID-19 measures 0.82 (0.80–0.84)  Netherlands  During COVID  0.84 (0.82–0.86)  before COVID  0.85 (0.83–87) |
| 7.Bonichini & Tremolada, 2021 | Italy, Cross sectional | Italian population, 1839 | Italians, 77.8% of female | GH12 (General Health Questionnaire) | Pearson’s correlations were run to verify links between normally distributed variables  ANOVAs were employed to assess the difference in dependent variables, according to independent variables  Hierarchical regression models were run to identify the socio-demographic, daily routine and emotions factors impacting on GH12 scores as the dependent variables.  A p-value < 0.05 for significance | The mean GH12 score in participants amounted to 17.86, with an SD of 5.85, reflecting a contingent moderate stressful impact on QoL  GH12 identified 39% of respondents as having subclinical QoL scores (score ≥ 15)  24.5% of such respondents as having very problematic scores (score ≥ 19), and 36.5% of such respondents as having normal scores (score < 15)  ANOVAs showed there was a significant difference (F(2, 1.836) = 5.50, p = 0.004, η 2 = 0.01) in mean GH12 scores |
| 8. Xiaoxiao Chen et al. 2021 | China, cross-sectional | Chinese peoples, 2420 | Chinese peoples,49.1 % female | EQ-5D and VAS | To compare EQ-5D between Deqing and Taizhou using the independent-samples t tests and the χ2 tests, or Wilcoxon and Kruskal-Wallis test when data distribution was skewed  Correlation between VAS and EQ-5D values was evaluated with the Pearson correlation coefficient  Multiple linear regression analysis was performed to assess the associations  Coefficient (β) and 95% confidence intervals (CIs) were calculated | The mean EQ-5D score and VAS were 0.990 and 93.5  Multiple linear regression showed that the quality of life measures was related to physical activities (β = 0.006) and keeping home ventilation (β = 0.063) in Deqing, and were related to wearing a mask when going out (β = 0.014), keeping home ventilation (β = 0.061), other marital status (β = − 0.011), worry about the epidemic (β = − 0.005) and having a centralized or home quarantine (β = − 0.005) in Taizhou |
| 9. Hang Choi et al 2021 | Hong Kong, Cross sectional | Hong Kong people,  500 | Hong Kong, 54.8% female | WHOQOL-BREF | Mean and SD scores of the WHOQOL-BREF were calculated  Independent t-tests were used to compare the mean HRQoL score of four domains between two groups (“yes” vs. “no”)  Cohen’s d was also calculated, which classifies effect sizes as small (d = 0.2), medium (d = 0.5), and large (d = 0.8)  Multiple linear regression analysis was used to test the robustness of the relationship | 69.6% were worried about contracting COVID-19, and 41.4% frequently suspected themselves of being infected  29.0% were concerned by the lack of disinfectants  All of these findings were associated with poorer HRQoL in the physical and psychological health, social relationships, and environment domains  47.4% of participants were concerned that they may lose their job because of the pandemic  39.4% were bothered by the insufficient supply of surgical masks |
| 10. Epifanio et al. 2021 | Italy, cross sectional | Italian population, 2251 | Italy 74 % female | WHOQOL-BREF | Pearson’s χ 2 test and Student’s t test for independent samples for nominal and continuous demographic variables were used, respectively  Analysis of variance (ANOVA) was used to analyze the differences in respondents’ levels of QoL at the global score of the WHOQOL-BREF  Multivariate analysis of variance (MANOVA) was employed to analyze the differences in levels of QoL at domain scores of the WHOQOL-BREF | There was a Significant difference in QoL depending on a number of variables, including sex, area of residence in Italy, and being diagnosed with a medical/psychiatric condition  The overall average score at the WHOQOL-BREF was 54.48 (SD = 7.77)  The item with the lowest scores was 14 (about the use of spare time), given that 932 (41.4%) participants reported to have little or no time for leisure at the time of data collection  Regarding the other three domains of the WHOQOL, items with lowest scores were: item 15 for the physical domain, as 1019 (45.3%) participants reported little or no possibility to do physical activity; item 5 for the psychological domain, with 712 (31.6%) respondents reporting that they were not enjoying their lives at the time of data collection, and item 21 for social relationships, as 843 (37.4%) respondents reported that they were little or not at all satisfied with their sexual life |
| 11. Ferreira et al. 2021 | Portugal, cross sectional | Portugal, 904 | Portugal, female 72.9% | EQ-5D-5L | EQ-5D-5L values were compared to the Portuguese population’s norms, for which EQ-5D-5L data were collected in the aforementioned cross-sectional study  Correlations between the EQ-5D-5L and GAD-7 scores were evaluated using Spearman’s rank correlation coefficient  The Mann–Whitney U and Kruskal–Wallis H tests were the most appropriate in this context  Linear model (GLM) with a Poisson distribution and log link was also used to examine determinants of HRQoL during the COVID-19 | Results suggest that those quarantined at home experienced higher levels of anxiety and a lower HRQoL compared with the pre-COVID-19 pandemic population  Females and elderly individuals experienced the highest levels of anxiety and poorest HRQoL |
| 12.Khodami et al. 2020 | Africa, North America, Asia, Australia, Europe, South America, Cross sectional study | Africa (554), North America (497), Asia (881), Australia (494), Europe ( 527 ), South America (119), 3002 part | Africa (Female: 8.9%), North America (female: 8.1%), Asia (14.2% ), Australia ( 7.9%), Europe ( 9%), South America ( 2.2%), 3002 | COVID-19 QoL questionnaire | The steadfastness of the applied scales was evaluated using Cronbach’s alpha  A Binary forward regression has been executed to predict the prevalence of behavioral impairments and Covid-19 QoL among all users. | The results showed that Quality of life was significantly decreased over time,  perceived stress level is raised significantly  and an increased level of difficulty in emotion regulation has happened  Younger peoples and individuals who had a worsening quality of life response tended to show more stress and emotion regulation problems |
| 13. Lipskaya-Velikovsky (2021) | Israel, Cross sectional | Israel, 571 | Israel, Female, 77.2% | WHOQOL-BREF | The type of distribution was detected using the Kolmogorov–Smirnov test  To estimate between-group differences, we used an independent samples t-test and one-way ANOVA or Mann–Whitney and Kruskal–Wallis tests  For each aspect of QOL, a multivariate model of explanation was built, using linear regression with a stepwise method  The following criteria were used to evaluate fitting of the models to the data: (1) A non-significant Chi-square p-value (p > 0.05); (2) a comparative fit index (CFI) value greater than 0.90; (3) a Tucker–Lewis Index (TLI) value greater than 0.90; and (4) a root mean square error of approximation (RMSEA) value less than 0.05 | The COVID-19 has had a wide impact on the general population, with the potential for negative secondary impacts  Women, young adults, and the unemployed are at high risk for secondary effects (ORs not available) |
| 14. Iglesias-López et al 2021 | Spain, cross sectional | Spain, 225 | Female,70.22% | SF-36 (SF-36v2) | Mean and standard deviation for the quantitative variables were calculated  Bivariate logistic regression with the dichotomous variables and an ordinal logistic regression with the categorical variables to report odds ratios (OR), in order to carry out a predictive analysis  Pearson chi-square was also used to relate dichotomous and categorical variables in a transversal analysis | The presence of pain in subjects undergoing confinement is persistent, with varying intensity and frequency based on age, gender, physical activity, and work status  In any of these conditions, the quality of life of the subjects in confinement has been severely affected |
| 15. Ping et al. 2020 | China, Cross sectional | China, 1139 | Female, 59.6% | EQ-5D | Mean and SD were calculated for continuous variables, frequencies and percentages for categorical variables  The relationships of all factors and the scores of EQ-5D were analyzed with t-test, analysis of ANOVA and nonparametric statistics (Mann-Whitney U test or Kruskall-Wallis test)  Fisher‘s exact test was used when exact theory frequency less than 1  Logistic regression model was used to the five health dimensions as dependent variables  Statistical significance was set at 0.05 using two-side tests. | The risk of pain/discomfort and anxiety/depression in general population in China raised significantly with aging, with chronic disease, lower income, epidemic effects, worried about get COVID-19 during the COVID-19 pandemic |
| 16. Qi et al. 2020 | China, Cross sectional study | China, 645 | Female, 61.2% | SF-8 (Chinese version) | Associations between physical activity, HRQoL, and perceived stress with demographic characteristics were computed using Pearson’s r, paired t-test, and analysis of variance (ANOVA)  The significant level was set at 0.05. | Participants’ average physical component summary score (PCS) and mental component summary score (MCS) for HRQoL were 75.3 (SD = 16.6) and 66.6 (SD = 19.3), respectively.  More than half of participants (53.0%) reported moderate levels of stress  Significant correlations between physical activity, HRQoL, and levels of perceived stress were observed (p < 0.05)  Prolonged sitting time was also found to have a negative effect on HRQoL (p < 0.05) |
| 17. Xuan Tran et al. 2020 | Vietnam, cross sectional | Vietnam, 341 | Vietnam, 65.7% female | EQ-5D-5L and EQ-VAS | We used t-test or Mann Whitney test for ordinal variables and Fisher-exact test or Chi-square test for nominal variables to compare groups.  Ordered logistic regression was employed to examine factors correlated with the impact of COVID-19 on respondents’ family income  A multivariable Tobit regression model was applied to identify factors associated with HRQOL score  Significance P-value<0.05 | 66.9% reported household income loss due to the impact of COVID-19  People holding undergraduate degrees, working in other sectors rather than healthcare, and having definite-term contract had a higher likelihood of income reduction  The mean score of EQ-5D-5L and EQ-VAS was 0.95 (± 0.07) and 88.2 (± 11.0), respectively  The domain of Anxiety/Depression had the highest proportion of reporting any problems among 5 dimensions of EQ-5D-5L (38.7%)  Being female, having chronic conditions and living in the family with 3–5 members were associated with lower HRQOL score |
| 18.Vitorino et al. 2021 | Brazil, Cross sectional | Brazil, 1156 | Brazil, 69.6% female | WHOQOL-BREF | Means and SD were used, (95% CI)  Student’s t-test and one-way ANOVA were used to compare the dependent variable means  Pearson’s correlation was performed between continuous independent variables  Logistic regression models were used to explore the associations between independent variables and significant depressive symptoms and significant anxiety symptoms  Multivariate general linear models (GLM) were used to assess the effects of the independent variables  Multivariate logistic regression models and GLM included only those independent variables that reached a P < 0.10 in bivariate analyses  Significance at P < 0.05 (two-tailed) | Scores on the social relationships QoL domain were lower among participants who had a family member or friend with COVID-19 and among those who engaged in negative forms of SRC  The quarantine during the COVID-19 pandemic has limited personal contact with family and friends, adversely affected sexual activity, and has restricted other activities that are assessed in the social relationships QoL domain.  positive forms of SRC were associated with better scores on this domain, as reported in other studies  Healthcare professionals had better outcomes on environment QoL, whereas participants who had a friend or family member with COVID-19 had worse scores on this domain  Healthcare professionals in Brazil are not subject to the quarantine because they must continue to care for people, and as noted earlier have higher incomes and better circumstances |
| 19. Mai Quynh Vu et al. 2020 | Vietnam, Cross sectional | Vietnam, 406 part | Vietnam, 69.7% female | EQ-5D-5L | In Government quarantine facilities" and "Self-isolation at private place" were small, and the score of EQ-5D-5L and EQ-VAS were not normally distributed, the Mann–Whitney U tests and Kruskal Wallis tests were carried out to identify the difference in HRQOL among participants.  The Dunn’s tests were used as the Poc-hoc analysis of the Kruskal-Wallis to examine the differences among multiple pairwise comparisons  Kruskal Wallis test was employed to test the hypothesis. Significance P<0.05 | The mean EQ-VAS was reported the highest at 90.5 (SD: 7.98) among people in government quarantine facilities, followed by 88.54 (SD: 12.24) among general population and 86.54 (SD 13.69) among people in self-isolation group. EQ-5D-5L value was reported the highest among general population at 0.95 (SD: 0.07), followed by 0.94 (SD: 0.12) among people in government quarantine facilities, and 0.93 (SD: 0.13) among people who did self-isolation. Overall, most people, at any level, reported having problems with anxiety and/or depression in all groups |
| 20. Anne Yee et al. 2021 | Malaysia, Web based cross sectional | Malaysia, 528 part | Malaysia, 62% | WHOQOL-BREF | Bivariate analyses were used to assess the association between sociodemographic data, COVID19-related variables, and coping strategies (Brief-COPE) with depression  Chi-squared test was used for categorical variables, Fisher’s exact test was used for categorical variables with 2×2 contingency table and one of the expected value being less than 5, and independent t test for continuous variables  The effect size was presented in phi for categorical variables, in which 0.2–0.29=weak, 0.30–0.39=moderate, 0.40– 0.69=strong, 0.70 or higher=very strong; and Cohen’s d for continuous variables, in which 0.20–0.46=small, 0.50–0.79=medium, and 0.80 or higher=large  Logistic regression method was used to analyze the significantly differences  The relationship between each domain of WHOQOL-BREF as dependent variables and mild-to-severe depression as independent variable, while controlling the significant variables in logistic regression as covariates, was explored using Analysis of Covariance (ANCOVA)  Significance<0.05 | Our study highlighted that approximately one in three individual experienced mild-to-severe depression during the nationwide MCO. The varied impact of the pandemic on mental health could be due to different population characteristics and coping strategies used. Identifying  those at higher risk to develop depression during MCO for COVID-19 pandemic could help mental healthcare service providers to plan services for those susceptible, thereby mitigating the pandemic’s effect on quality of life. |
| 21. Campbell and Davison,2021 | Scotland, mixed method sequential study | Scotland population, 267 part | Female: 92% | EQLS | A descriptive and correlational analysis were conducted to explore the association between scales  A multiple regression analysis was undertaken to explore the extent of these associations and determine if these correlations were statistically significant. | Table 7 shows there are a strong relationship between HRQoL and Income, disability and living arrangement as well as social isolation and Disability and living arrangement  Correlation and multiple regression analyses showed a strong relationship between social isolation, gratitude, uncertainty and HRQoL, with social isolated being a significant predictor  Evidence of a strong correlation amongst disabled and low-income participants experiencing higher levels of social isolation was found  Uncertainty and levels of gratitude were also found to be correlated with social isolation and HRQoL |
| 22. Tonya Cross Hansel et al 2022 | USA,  Cross sectional | USA, 296 part | Female, 85% | WHOQOL-BREF | McNamara Chi square analyses were conducted to compare current cut-of scores or alcohol misuse cutoff with previous mental health and substance use problems  One sample Z tests were used to compare participant cut-of scores with 2019 population estimates  Zero order (Pearson product moment) correlations were conducted to assess associations among variables  The structural model was tested using SPSS analysis of moment structure (AMOS) version 27  Assumptions of normality and linearity were met; missing data was less than 5% and imputed using linear interpolation  Significant zero-order correlation paths were added to the model but did not reveal good ft (RMSE>0.05) | Most would expect quality of life to be challenged during a global pandemic  Both past and current mental health were strong predictors of quality of life  COVID-19 experience  role in quality of life, where participants who felt socially isolated reported personal health effects, or COVID-19 suspected or diagnosed participants reported lower quality of life  Results demonstrated that higher individual and community stressors result in poor mental health and inadvertently decrease quality of life |
| 23. Shorouk Mohsen et al. 2022 | Egypt, community based cross sectional study | Egypt, 500 participants | Egypt, 44.4% female | COV19- Impact on Quality of Life | Categorical data were presented as numbers and percentages of the total, while continuous data were expressed as mean±standard deviation  Student’s t-test was used to compare normally distributed continuous data between 2 groups  Stepwise multiple linear regression analysis was applied to evaluate the contribution of factors found to be significant in bivariate analysis in predicting QoL and COVID-19 total score | Total COV19-QoL scale score (mean±standard deviation) was 2.3±0.6  Two items show the highest mean with 2.6±0.7 (quality of life in general and perception of danger on their personal safety) indicating the poorest quality of life regarding these 2 items  The lowest mean score is related to the perception of mental health deterioration (1.9±0.8)  sex (regression coefficient (95% CI)=0.1 (0.02 to 0.2), p value=0.02), monthly income (regression coefficient (95% CI)=0.1 (0.004 to 0.2), p value=0.04), knowing someone infected with COVID19 (regression coefficient (95% CI)=0.15 (0.08 to 0.3), p value=0.001), and data collection time (regression coefficient (95% CI)=0.1 (0.006 to 0.2), p value=0.04) were the independent predictors for overall QoL scale score |
| 24. Samlani Z1 et al. 2020 | Morocco, cross sectional study | Morocco, 279 participants | Morocco peoples, 48.4% female | SF12 | t-student test was used to compare the means of the summary physical and mental scores of the SF-12, after having checked the normality of the distribution of the two scores The materiality threshold was set at 0.05 | participants obtained a total average score of 70.60 (±13.1) with a mental health score (MCS) of 34.49 (±6.44) and a physical health score (PCS) of 36.10 (± 5.82)  Physical (PCS) and mental (MCS) scores of participants with chronic diseases were 32.51 (±7.14) and 29.28 (±1.23), respectively  Overall, the participants’ PCS and MCS scores suffered from chronic diseases and the elderly participants were lower than those of young participants without comorbidities |
| 25.Teotônio et al 2020 | Brazilia, cross sectional | Brazilia, 1859 participants | Brazilian peoples, 72.56% | WOHQoL-BREEF | one-way repeated measures ANOVA followed by Bonferroni’s post-hoc tests. A Student’s t-test and Variance Analysis (ANOVA) followed by Tukey post-hoc analysis was used to compare the domains’ values with the socioeconomic and demographic variables. | Mean total score 62.59 (11.54)  Psychological 15.54 (3.81)  Social 14.79 (3.40)  Physical 17.62 (2.97)  Economic 14.64 (4.96)  Females presented worse QoL than males (p < 0.05)  Individuals age ≥ 40 y/o presented better QoL for all domains  Individuals with partners presented better QoL scores  White individuals presented better QoL scores than brown and black respondents  Higher educational level was associated with better QoL  Unemployed individuals presented lower QoL scores  Individuals that tested positive for COVID-19 presented the lowest QoL  QoL was affected when a family member was diagnosed with COVID-19 |

EQLS: European Quality of Life Survey, EQ-5D/EQ-5D-5L: EuroQoL-Five dimensions, WHOOoL-BTREF: World Health Organization Quality of Life BREF; SF12: Short form; COVID-19 QoL questionnaire: COVID-19 Quality of Life Questionnaire; GH12: The General Health Questionnaire; MLT: My Life Today Questionnaire
