# Supplementary material for: Impact of COVID-19 on health-related quality of life in the general population: A systematic review and meta-analysis
Source: PLOS Glob Public Health. 2023 Oct 26;3(10):e0002137. doi: 10.1371/journal.pgph.0002137 (PMC10602258; doi:10.1371/journal.pgph.0002137)
Supplement: S1 Text — (DOCX) [file pgph.0002137.s003.docx]

**S3: Search string**

“ ("Quality of Life"[Mesh] OR “quality of life” [tw] OR **“**Health-related Quality of Life” [tw] OR HRQoL[tw]) AND **(**"COVID-19"[Mesh] OR COVID-19[tw] OR “SARS-CoV-2” [tw] OR Sars-cov-2[tw] OR Coronavirus[tw] OR SARS OR “Coronavirus disease 2019” [tw] OR “severe acute respiratory syndrome coronavirus 2” [tw] OR “2019-nCoV Infection” [tw] OR 2019-nCoV[tw] OR “COVID-19 Virus Disease” [tw]) AND **(**"Population"[Mesh] OR “general population”[tw] OR “general public”[tw] OR “public”[tw] OR “communit*”[tw])
