# Supplementary material for: Impact of COVID-19 on health-related quality of life in the general population: A systematic review and meta-analysis
Source: PLOS Glob Public Health. 2023 Oct 26;3(10):e0002137. doi: 10.1371/journal.pgph.0002137 (PMC10602258; doi:10.1371/journal.pgph.0002137)
Supplement: Attachment — Submitted filename: PGPH-D-23-00668_AnilEdit.pdf [file pgph.0002137.s004.pdf]

PLOS Global Public Health

Impact of COVID-19 on Health-Related Quality of Life in the General Population: A Systematic Review

--Manuscript Draft--

**Manuscript Number:**	PGPH-D-23-00668
**Article Type:**	Research Article
**Full Title:**	Impact of COVID-19 on Health-Related Quality of Life in the General Population: A Systematic Review
**Short Title:**	Impact of COVID-19 on Quality of Life in the General Population
**Corresponding Author:**	Desire Aime Nshimirimana, MBChB,Msc<br>Mount Kenya University<br>Thika, KENYA
**Order of Authors:**	Desire Aime Nshimirimana, MBChB,Msc
	Donald Kokonya, MBChB, MMed
	Jesse Gitaka, MBChB,PhD
	Bernard Wesonga, MBChB, PhD
	Japheth Nzioki Mativo, Msc, PhD
	Jean Marie Vianey Rukanikigitero, MBChB,MPH, MMed
**Keywords:**	Health Related Quality of Life, HRQoL, Quality of Life, QoL, COVID-19, General population
**Abstract:**	Background: The World Health Organization declared coronavirus disease of 2019 as an epidemic and public health emergency of international concern on January 30th, 2020. Different factors can affect quality of life during a pandemic. Quality of life is considered multidimensional and subjective and it is assessed by patients using the patient reported outcome measures. The objective of this review is to assess the impact of coronavirus disease of 2019 and associated factors on the Quality of Life in the general population. Methods: This review was conducted and reported according to the Preferred Reporting Items for Systematic Reviews and Meta-Analyses. A protocol was registered in the international Prospective Register of Systematic Reviews database(CRD42021269897). A comprehensive electronic search in PubMed, EBSCO Host Research Databases, MEDLINE and Google scholar search engine was conducted. Results: A total number of 1,7000,074 articles were identified from electronic databases. 25 full text papers were retained for qualitative synthesis. Seven main quality of life scales were used to assess the quality of life of the general population; World Health Organization Quality of Life-bref, EuroQuality of Life-Five dimensions, Short Form, European Quality of Life Survey, coronavirus disease of 2019 Quality of Life, General Health Questionnaire12 and My Life Today Questionnaire. The mean World Health Organization Quality of Life-bref was found to be 53.38% 95% confidence interval [38.50-68.27] and EuroQuality of Life-Five dimensions was 0.88 95% confidence interval [0.69-1.07]. Several factors have been linked to the Coronavirus disease of 2019 impact on quality of life such as sociodemographic factors, peoples living with chronic diseases, confinement and financial constraints. Conclusion: This review confirms that the Coronavirus disease of 2019 pandemic affected the quality of life of the general population worldwide. Several factors such as sociodemographic, peoples living with chronic diseases, confinement and financial constraints affected the quality of life.
**Opposed Reviewers:**	
**Additional Information:**	
**Question**	**Response**
**Financial Disclosure**	This study did not receive any grant/financial aid from who ever

Powered by Editorial Manager® and ProduXion Manager® from Aries Systems Corporation

Enter a financial disclosure statement that describes the sources of funding for the work included in this submission and the role the funder(s) played. This includes grants and any commercial funding of the work or authors.<br><br>This statement will be typeset if the manuscript is accepted for publication.<br><br>*Please review the submission guidelines and the instructions link below for detailed requirements and guidance.*	
**Competing Interests**<br><br>On behalf of all authors, disclose any competing interests that could be perceived to bias this work.<br><br>This statement will be typeset if the manuscript is accepted for publication.<br><br>*Please review the instructions link below and PLOS Global Public Health's competing interests policy to determine what information must be disclosed at submission.*	Authors declare no conflict of interest
**Data Availability**<br><br>Before publication, Authors are required to make fully available and without restriction all data underlying their findings. Please see our PLOS Data Policy page for detailed information on this policy.<br><br>A **Data Availability Statement**, detailing where the data can be accessed, is required at first submission. Insert your Data Availability Statement in the box below.	A protocol was registered in the international Prospective Register of Systematic Reviews database (PROSPERO) registration number (CRD42021269897)

Please see the data reporting section of our submission guidelines for instructions on what you need to include in your Data Availability Statement.<br><br>This statement will be typeset if the manuscript is accepted for publication.<br><br>*PLOS allows rare exemptions to address legal and ethical concerns. If you have legal or ethical restrictions, please use the box below to detail these in full sentences for the Journal team to consider.*	

Powered by Editorial Manager® and ProduXion Manager® from Aries Systems Corporation

Chief Editor

PLOS ONE Global Public Health

April 10th, 2023

Dear Chief Editor,

I hereby submit the manuscript entitled "Impact of COVID-19 on Health-Related Quality of Life in the General Population: A Systematic Review" to be considered for publication as a research article in the PLOS ONE journal. There is a gap regarding the impact of COVID-19 on the Health Related Quality of Life (HRQoL) in the general population. The objective of this study is to assess the impact of COVID-19 and its related factors on the HRQoL in the general population. Our results show that COVID-19 affected negatively the quality of life of the general population and identified some factors related factors. The findings of our study will help decision-makers, Healthcare managers and clinicians to plan for prevention of low quality of life for future pandemics. The study did not get any fund from whichever source and there was no conflict of interest declared. The study is not being considered elsewhere and has not been published. All authors have consented to the submission of the manuscript. The submitted file has the following attachments; main manuscript, supplementary documents, cover letter and supplementary figures. We believe the manuscript is suitable for publication in your reputed journal.

Your faithfully,

Desire Aime Nshimirimana

Manuscript

Click here to access/download;Manuscript;Manuscript HRQoL.docx

Impact of COVID-19 on Health-Related Quality of Life in the General Population: A Systematic Review

*Desire Aime Nshimirimana[1,2,5], Donald Kokonya[2], Jesse Gitaka[1], Bernard Wesonga[2], Japheth Nzioki Mativo[3], Jean Marie Vianey Rukanikigitero[4]

Affiliation

[1]Departement of Research & Innovation, Mont Kenya University, Thika, Kenya

[2]Department of Community Health & Behavioral Sciences, School of Medicine, Masinde Muliro University of Science & Technology, Kakamega, Kenya

[3]Department of Environmental Health, Colleges of Health Sciences, Jumeira University-Dubai, The United Arab Emirates

[4]Department of Dermatology, International Hospital Kampala, Uganda

[5]Department of Health Systems Management, School of Health Sciences, Nairobi Campus, Kenya Methodist University (KeMU)

Corresponding author: desireaime.da@gmail.com

Abstract

Background: The World Health Organization declared coronavirus disease of 2019 as an epidemic and public health emergency of international concern on January 30th, 2020. Different factors can affect quality of life during a pandemic. Quality of life is considered multidimensional and subjective and it is assessed by patients using the patient reported outcome measures. The objective of this review is to assess the impact of coronavirus disease of 2019 and associated factors on the Quality of Life in the general population. **Methods:** This review was conducted and reported according to the Preferred Reporting Items for Systematic Reviews and Meta-Analyses.

A protocol was registered in the international Prospective Register of Systematic Reviews database(CRD42021269897). A comprehensive electronic search in PubMed, EBSCO Host Research Databases, MEDLINE and Google scholar search engine was conducted. **Results:** A total number of 1,7000,074 articles were identified from electronic databases. 25 full text papers were retained for qualitative synthesis. Seven main quality of life scales were used to assess the quality of life of the general population; World Health Organization Quality of Life-bref, EuroQuality of Life-Five dimensions, Short Form, European Quality of Life Survey, coronavirus disease of 2019 Quality of Life, General Health Questionnaire12 and My Life Today Questionnaire. The mean World Health Organization Quality of Life-bref was found to be 53.38% 95% confidence interval [38.50-68.27] and EuroQuality of Life-Five dimensions was 0.88 95% confidence interval [0.69-1.07]. Several factors have been linked to the Coronavirus disease of 2019 impact on quality of life such as sociodemographic factors, peoples living with chronic diseases, confinement and financial constraints. **Conclusion:** This review confirms that the Coronavirus disease of 2019 pandemic affected the quality of life of the general population worldwide. Several factors such as sociodemographic, peoples living with chronic diseases, confinement and financial constraints affected the quality of life.

Keywords: Health Related Quality of Life, HRQoL, Quality of Life, QoL, COVID-19, General population

Background

The World Health Organization (WHO) declared coronavirus disease of 2019 (COVID-19) as an epidemic and public health emergency of international concern on January 30th, 2020. The virus is known to have originated from Wuhan City, Hubei Province, China from December 2019. COVID-19 drew global attention due to rapid increase in the numbers reported both in China and

internationally within shortest period (1) and by February 20th, 2020, the number of contaminated cases in China reached a cumulative total of 75,465 cases of COVID-19 and it had already spread to more than 25 countries, among them Germany, Italy, France, Japan, Malaysia, Singapore, South Korea, Spain, Thailand, Vietnam, the United Arab Emirates, the United Kingdom (UK), the United States of America (USA) and Africa(2). According to WHO (2021), by the 19th July 2021, a total of 190,597,409 confirmed cases of COVID-19, among them 4,093,145 deaths and only 3,430,051,539 vaccine doses had already been administered by 18th July 2021 Worldwide. Psychological conditions such as depression, anxiety, and stress can contribute to the deterioration of quality of life (QoL) of populations (3). Health related quality of life (HRQoL) is considered multidimensional and subjective and it is assessed by patients using patient reported outcome measures (PROMs). According to the WHO, the HRQoL is defined as the general perception of individuals of their positions in life (i) considering, the culture and value systems and (ii) in relation to expectations, goals, standards, and concerns(4). The HRQoL considers a wide-ranging concept influenced in a complex and interconnected manner by the psychological state, physical health, personal beliefs, social relationships and relationship to prominent features of the environment (5). A systematic review by Nobari et al (2021) discussed the impact of COVID-19 on the HRQoL on children and adolescents due to a multitude of changes in their lives, including wearing masks, school closures, home confinements and social distancing rules, which can burden them substantially. Their results showed that lockdown significantly affected the HRQoL, happiness and optimism ($p < 0.001$), as well as perceived stress. In their findings, the authors reported that 15.3% (n = 146) of children and adolescents had low HRQoL before COVID-19 outbreak and during the pandemic, 40.2% of them reported low HRQoL (6). In the Kingdom of Saudi Arabia (7) assessed the quality of life during COVID-19 in the general population and found out that being

male (OR = 1.96; 95% CI = 1.31–2.94), aged between 26 to 35 years (OR = 5.1; 95% CI = 1.33–19.37), non-Saudi participants (OR = 1.69; 95% CI = 1.06–2.57), individuals with chronic diseases (OR = 2.15; 95% CI = 1.33–3.48), loss of job (OR = 2.18; 95% CI = 1.04–4.57) and participants with depression (OR = 5.70; 95% CI = 3.59–9.05), anxiety (OR = 5.47; 95% CI = 3.38–8.84) and stress (OR = 6.55; 95% CI = 4.01–10.70) were at a high risk of having lower levels of QoL during the COVID-19 pandemic and lockdown period (7). Persson et al (2020) assessed the change in the quality of life (QoL) of the Swedish population using data of February and April 2020 and found out that on visual analogue scale (VAS), the mean QoL reduced from 77.1(SD:17.7) in February to 68.7(SD:68.7) in April 2020, a reduction of 8.4% pre and post pandemic measurements (P<0.000)(8). Matos et al (2021) compared the quality of life (QoL) of Brazilian dietitians before SARS-COV-2 (3.83 ± 0.59) and during the pandemic (3.36 ± 0.66) and they find that data was statistically different. By comparing prior and during the COVID-19 pandemic, all variables and domains presented statistical differences (better before the pandemic period) and the psychological health domain was the most affected and concluded that SARS-COV-2 pandemic negatively impacted the QoL of Brazilian dietitians (9). To the best of our knowledge, this is one of the first systematic reviews, assessing the impact of COVID-19 on the HRQoL in the general population, based on the objective of this systematic review to assess the impact of COVID-19 and associated factors to Health Related Quality of Life in the general population.

Methods

Design and protocol

This systematic review was conducted and reported according to the Preferred Reporting Items for Systematic Reviews and Meta-Analyses (PRISMA)(10). A protocol was registered in the

international Prospective Register of Systematic Reviews database (PROSPERO) registration number (CRD42021269897)

Eligibility

Articles were included if they were (i) primary and empirical studies, quantitative, cross-sectional, cohort and case-control studies, peer reviewed, assessing effects of COVID-19 on the quality of life before and during COVID-19 in the general population, they utilized validated scales for measurement, published in English language with no date limit from inception to June 30th, 2022. Were excluded if (ii) focusing on subgroups of populations (i.e health care workers, population with previous mental health, population with cancer, HIV or any other chronic disease), secondary and non-empirical, non-peer reviews, review articles (Scoping, narrative and Systematic reviews), papers on Medrxiv and SSRN server, comments, letters, conference abstracts, books and book chapters, articles which don't assess the quality of life, papers on the population with previous mental health problems or papers not assessing quality of life in the general population during the COVID-19 pandemic. There was no limit on the number of papers to synthetize and all the studies, which satisfied the eligibility criteria were included. Grey literature was used only to support the background section of the study.

Search strategy and selection

A comprehensive electronic search in the PubMed, EBSCO Host Research Databases (Academic Research Complete), MEDLINE (OVID) and Google scholar search engine was conducted from 5th January 2022 to 28th February 2022 and updated on 30th June, 2022. The search strategy and data extraction were designed by DAN, DK and JG using Medical Subject Headings (MeSH), field tags and the relevant keywords related to quality of life, COVID-19 and general population. Boolean operators, the thesaurus, truncation, nesting and quotation marks were also used where

applicable to strengthen the search. The full search strategy was provided in supplementary documents. Additional search of the references of retrieved systematic reviews through snow balling was performed as well. All the retrieved papers were downloaded and saved to Mendeley for text citations and referencing. The following was used as search string for PubMed; “ ("Quality of Life"[Mesh] OR “quality of life” [tw] OR **“**Health-related Quality of Life” [tw] OR HRQoL[tw]) AND **(**"COVID-19"[Mesh] OR COVID-19[tw] OR “SARS-CoV-2” [tw] OR Sars-cov-2[tw] OR Coronavirus[tw] OR SARS OR “Coronavirus disease 2019” [tw] OR “severe acute respiratory syndrome coronavirus 2” [tw] OR “2019-nCoV Infection” [tw] OR 2019-nCoV[tw] OR “COVID-19 Virus Disease” [tw]) AND **(**"Population"[Mesh] OR “general population”[tw] OR “general public”[tw] OR “public”[tw] OR “communit*”[tw])

Data extraction technique

A standardized data collection tool to extract relevant information from papers was designed. The following data was collected; authors, country of publication, study design, sample size, demographic characteristics, HRQoL before COVID-19, HRQoL during or after the COVID-19, HRQoL measurement tool, statistical tests and risk factors as well as their odds ratios (OR). Data was extracted by two authors (DAN & BW) and verified by the second author (JNM). Discrepancies were resolved by the 3rd author (JG).

Quality appraisal

The two authors (DAN and JG) independently assessed the quality of the papers included, using a modified Newcastle-Ottawa Scale (NOS) which was modified accordingly for cross-sectional studies. The following items were the quality criteria used in cross-sectional studies and for other study designs and this was updated accordingly: sample representation, sample size, response rate and validated measurement tools with appropriate cut-off points and the control of confounding

variables or use of multiple regression. The quality score ranged between 0 and 5 and any study scoring > or =3 was considered as high and any study scoring < 3 was considered to be at low quality.

Main outcome

Health related quality of life (HRQoL)

Measures of effect

Health related quality of life measurements such as means of EQ5D and WHOQoL-BREF were calculated as well as their standard deviations

Heterogeneity and risk of bias assessment of included studies

The risk of bias was assessed for EQ5D and WHOQoL_BREF papers because many studies were performed and there was a need to check for risk of bias, heterogeneity and mean effect size if any. Other studies were not allowing for the same because they were performed more or less once with the same instruments. A random-effects model was fitted to the data. The amount of heterogeneity (i.e., τ^2), was estimated using the restricted maximum-likelihood estimator (11). In addition to the estimate of τ^2, the Q-test for heterogeneity (12) and the I^2 statistic(13) are reported. In case any amount of heterogeneity is detected (i.e., $\hat{\tau}^2 > 0$, regardless of the results of the Q-test), a prediction interval for the true outcomes is also provided(14). Studentized residuals and Cook's distances are used to examine whether studies may be outliers and/or influential in the context of the model(15). Studies with a studentized residual larger than the $100 \times \big(1 - 0.05/(2 \times k)\big)$th percentile of a standard normal distribution are considered potential outliers (i.e., using a Bonferroni correction with two-sided $\alpha = 0.05$ for k studies included in the meta-analysis). Studies with a Cook's distance larger than the median plus six times the interquartile range of the Cook's distances are considered to be influential. The rank correlation test (16) and the regression

test(17), using the standard error of the observed outcomes as predictor, are used to check for funnel plot asymmetry. The analysis was carried out using R (version 4.2.1) (R Core Team, 2020) and the **metafor** package (version 3.8.1)(18).

Data synthesis

Data was collected on the authors, countries of publication, study designs, sample sizes, demographic characteristics, the HRQoL before, during or after COVID-19 pandemic, the HRQoL measurement tool, statistical tests and risk factors were summarized following the "Institute of Medicine committee on the standards for systematic reviews of comparative effectiveness research: Finding out what works in health care; standards for systematic reviews: recommended standards for qualitative synthesis" (50) and the key characteristics of included studies if similar were grouped, synthetized qualitatively and discussed in order to draw conclusions. The mean effect size was performed for both EQ5D and WHOQoL_BREF studies.

Results

A total number of 1,700,074 articles were identified from electronic databases search on PubMed (1,334,241), Medline (OVID) (365,401), EBSCO (Host Research Databases (Academic Research Complete)) (425) and manual search with Google scholar search engine (5). After removing duplicates and papers not related to quality of life (1,700,620) 546 were retained. 461 full text papers and abstracts were removed to retain 85 full papers for screening. Finally, 25 full text papers were retained for summarizing and synthesizing (PRISMA)(Fig1).

Characteristics of included studies

The total sample of included studies was N=22,967 participants and ranges from 225 to 3,002 participants. The majority (64.85%) were female (n=14,894). Among the papers, 3 studies were done in China(19,20, 21), 2 Morocco (22, 23), 2 Vietnam (24, 25), 2 Italy (26, 27), 1 Saudi Arabia (7), 1 Malaysia (28, 36), 1 Jordan (29), 1 Philippines (30), 1 Hong Kong (31), 1 Portugal (32), 1 Israel (33), 1 Spain (34), 1 Brazil (35), 1 Scotland (37), 1 USA (38), 1 Egypt (39), 1 study done in two countries Belgium and Netherlands (40) and one in Africa, North America, Asia, Australia, Europe, South America (41). Nine articles used the WHOQoL-BREF tool to measure the quality of life in their respective countries (7, 28, 29, 31, 27, 33, 35, 36, 38), six papers utilized the EQ-5D/EQ-5D-5L (22, 40, 19, 32, 24, 25), three used SF12/SF-8/ SF36(34, 23, 21), one utilized EQLS (37), one used GH12 (26), one utilized the COVID-19 QoL questionnaire (41), on used the COVID-19 (COV19- Impact on the quality of life (COV19-QoL) scale) (39) and one utilized MLT (30). The majority of the studies (n=23) were of cross-sectional design and only one (37) was of a mixed method. Nine studies utilized the WHOQoL-BREF (7, 28, 29, 31, 27, 33, 35, 36, 38), nine utilized the EQ-5D/EQ-5D-5L (22, 40, 19, 32,24, 25, 37), three articles utilized the SF12/SF-8/SF36 (34, 23, 21) one article utilized EQLS (37), one paper used GH12 (26). Another one assessed the HRQoL using COVID-19 QoL questionnaire (41) and one article used MLT (30) to assess HRQoL in general population.

Measurement tools

Eight (n=8) scales have been used to assess health related quality of life on the general population worldwide during COVID-19. EQ-5D: Euro_QoL-Five dimensions; is a preference and generic quality of life instrument to valuate and describe health related quality of life; the higher the index, the better the health. It describes health in terms of five dimensions; mobility, self-care, usual activities, pain/discomfort and anxiety/depression (42). The WHO_OoL-BREF: the WHO quality

of life BREF is a 26-item instrument with four domains: physical health (7 items), psychological health (6 items), social relationships (3 items), and environmental health (8 items) (43). SF12: Short form are generic health survey short-forms (don't use preference based approach) to assess quality of life which are used in research and clinical practice, health policy and general surveys (44). EQLS: European Quality of Life Survey is a 2012 scale which considers the following dimensions; employment and work-life balance, family and social life, health and public services, home and local environment, quality of society, social exclusion and community involvement, standard of living and deprivation, subjective well-being which is designed for the general population(45). GHQ: General Health Questionnaire is a measure of current mental health and since its development by Goldberg in the 1970s it has been extensively used in different settings and different cultures (46). COV19-QoL is a 6-item scale covering main areas of quality of life in relation to mental health. The first item covers patients' feelings about the impact of the current pandemic on their quality of life in general. The second and third ones include the participants' perceptions of possible mental and physical health deterioration. COV19-QoL scale is a recently developed specific reliable and valid tool assessing perceptions of deterioration in QoL as a result of the COVID-19 pandemic (39). MLT: My Life Today the 9-tem (4) scale was used to measure the participants' perceptions of various life domains, including the assessment of life in general population(30).

Table 1: Mean Scores of QoL reported in the included studies

Study	Measurement method	Mean QoL	Most affected domain	Least affected domain
1 Algahtani et al. 2020(7)	WHOQOL-BREF	39% (SD=NA)	Social-relationship	Environment
2 Abdullah et al. 2021(28)	WHOQOL-BREF	69.44% (SD=12.78)	Social relationship	Environment
3. Al-Shannaq et al 2020(29)	WHOQOL-BREF	73.21% (SD ¼ 16.17)	Social relationship	Environment

4 Aruta et al. 2021(30)	MLT	NA	NA	NA
5 Azizi et al 2020(22)	EQ-5D-5L	During confinement (0.86; P<0.0001 Before confinement (utility=0.91)	Mobility	Anxiety/depression
6 Ballegooijen et al 2021(40)	EQ-5D	0.79 (0.77–0.81) Belgium 0.84 (0.82–0.86) Netherlands	NA	NA
7 Bonichini & Tremolada, 2021(26)	GH12	17.86 (SD 5.85)	NA	NA
8 Chen et al. 2021(19)	EQ5D	0.990 (SD:N/A)	NA	NA
9 Choi et al 2021(31)	WHOQoL	61.685 % (SD=14.6275)	Physical health	Environmental health
10 Epifanio et al. 2021(27)	WHOQoL	54.48 % (SD = 7.77)	Physical domain	Environment
11 Ferreira et al. 2021(32)	EQ-5D-5L	0.861 (SD=0.027) (During COVID_19) 0.887 (SD=0.005) (pre-COVID_19)	Anxiety/depression	Self-care
12 Khodami et al. 2020(41)	COVID-19 QoL questionnaire	21(SD= 21.18) (5(SD: 4.789))	NA	NA
13 Lipskaya-Velikovsky (2021)(33)	WHOQoL	73.5 % (IQR:59.5-87.5) SD: NA	Physical	Environment
14 Iglesias-López et al 2021(34)	SF-36 (SF-36v2)	NA	Physical functioning	Mental health
15. Ping et al. 2020(20)	EQ-5D	0.949 (SD: 0.102) 85.52(SD: 19.37) VAS	Pain/disorder	Anxiety/depression
16 Qi et al. 2020(21)	SF8	75.3 (SD = 16.6)	physical	mental
17 Tran et al. 2020(24)	EQ-5D-5L and EQ-VAS	0.95 (± 0.07) and 88.2 (SD: ± 11.0)	Anxiety/depression	Self-care
18 Vitorino et al. 2021(35)	WHOQoL	14.325 (SD=2.8625)	Physical	anxiety
19 Quynh Vu et al. 2020(25)	EQ-5D-5L	0.95 (general pop)	NA	NA

20 Yee et al. 2021(36)	WHOQOL-BREF	13.2 (SD=2.8325)	Social relationship	Environment
21 Campbell & Davison,2021(37)	EQLS	NA	NA	NA
22 Hansel et al 2022(38)	WHOQOL-BREF	25.1 (SD=4.9)	NA	NA
23 Mohsen et al. 2022(39)	COV19-Questionnaire_Quality of Life	2.3 (SD=±0.6)	Quality of life in general and perception of danger	Perception of mental health perception
24 Samlani Z1 et al. 2020(23)	SF12	70.60 (SD: ±13.1)	Mental health	Mental health with chronic disease
25. Teotônio et al 2020(47)	WHOQoL-BREEF	62.59 (SD=11.54)	Economic	Physical

EQLS: European Quality of Life Survey, EQ-5D/EQ-5D-5L: EuroQoL-Five dimensions, WHO_OoL-BREF: World Health Organization Quality of Life BREF; SF12: Short form; COVID-19 QoL questionnaire; GH12: The General Health Questionnaire; MLT: My Life Today Questionnaire

Forest Plot WHO_BREF

A total of $k = 9$ studies were included in the analysis. The observed outcomes ranged from 13.2000 to 73.5000, with the majority of estimates being positive (100%). The estimated mean outcome based on the random-effects model was $\hat{\mu} = 53.3841$ (95% CI: 38.4992 to 68.2691). Therefore, the average outcome differed significantly from zero ($z = 7.0293$, $p < 0.0001$). A forest plot showing the observed outcomes and the estimate based on the random-effects model is shown (Fig2)

Fig3: Mean score using WHO_QoL-BREF

Study1 (7), study2 (28), Study3 (29), Study4 (31), Study5 (7), Study6 (33), Study 7 (36), Study8 (38), study 9 (47). The mean health related quality of life using WHOQoL_BREF is estimated at 53.38

Forest plot EQ5D

A total of $k = 8$ studies were included in the analysis. The observed outcomes ranged from 0.7900 to 0.9900, with the majority of estimates being positive (100%). The estimated average outcome based on the random-effects model was $\hat{\mu} = 0.8930$ (95% CI: 0.6566 to 1.1295).

Therefore, the average outcome differed significantly from zero ($z = 7.4024$, $p < 0.0001$). A forest plot showing the observed outcomes and the estimate based on the random-effects model is shown (Fig3)

Fig3: Mean score using EQ5D

Study 1(22), Study2 (40), Study3 (40), Study4 (19), Study5(32), Study6(20), Study7 (24), Study8 (25)

Heterogeneity and risk of bias of WHO-BREF studies

According to the Q-test, the true outcomes appear to be heterogeneous ($Q(8) = 656.8283$, $p < 0.0001$, $\hat{\tau}^2 = 509.6543$, $I^2 = 99.2019\%$). A 95% prediction interval for the true outcomes is given by 6.7003 to 100.0680. Hence, even though there may be some heterogeneity, the true outcomes of the studies are generally in the same direction as the estimated average outcome.

A funnel plot of the estimates is shown in Figure 5. The regression test indicated funnel plot asymmetry ($p = 0.0019$) but not the rank correlation test ($p = 0.1194$) (Fig4)

Fig4: WHOQoL_BREF Funnel plot

Study1 (7), study2 (28), Study3 (29), Study4 (31), Study5 (7), Study6 (33), Study 7 (36), Study8 (38), study 9 (47).

Heterogeneity and risk of bias of EQ5D studies

According to the Q-test, there was no significant amount of heterogeneity in the true outcomes ($Q(7) = 0.3149$, $p = 0.9999$, $\hat{\tau}^2 = 0.0000$, $I^2 = 0.0000\%$). A funnel plot of the estimates is shown in Figure 6. Neither the rank correlation nor the regression test indicated any funnel plot asymmetry ($p = 0.5664$ and $p = 0.8657$, respectively) (Fig5)

Fig5: EQ5D_QoL Funnel plot

Study 1(22), Study2 (40), Study3 (40), Study4 (19), Study5(32), Study6(20), Study7 (24), Study8 (25)

Table 2: Modified Newcastle-Ottawa quality assessment scale

Author & year of publication	Sample representativeness	Sample size (>500)	Response rate	Study used validate measurement tools with appropriate cut-offs	Control of confounding variables or used multiple regression	Score
1 Algahtani et al. 2020	+	+	-	+	+	4
2 Abdullah et al. 2021	+	-	+	+	+	4
3. Al-Shannaq et al 2020	+	-	+	+	+	4
4 Aruta et al. 2021	+	-	_	+	+	3
5 Azizi et al 2020	+	-	-	+	-	2
6 Ballegooijen et al 2021	+	+	-	+	-	3
7 Bonichini & Tremolada, 2021	+	+	+	+	+	5
8 Chen et al. 2021	+	+	-	+	+	4
9 Choi et al 2021	+	-	+	+	+	4
10 Epifanio et al. 2021	+	+	_	+	+	4
11 Ferreira et al. 2021	+	+	+	+	+	5
12 Khodami et al. 2020	+	+	-	+	+	4
13 Lipskaya-Velikovsky (2021)	+	-	_	+	+	3
14 Iglesias-López et al 2021	+		+	+	+	4
15. Ping et al. 2020	+	+	_	+	+	4
16 Qi et al. 2020	+	+	-	+	_	3
17 Tran et al. 2020	+	-	_	+	+	3
18 Vitorino et al. 2021	+	+	+	+	+	5
19 Vu et al. 2020	+	-	+	+	-	3

20 Yee et al. 2021	+	-	-	+	+	3
21 Campbell and Davison,2021	+	-	-	+	_	2
22 Tonya Cross Hansel et al 2022	+		_	+	+	3
23 Shorouk Mohsen et al. 2022	+	-	_	+	+	4
24 Samlani Z1 et al. 2020	+	-	_	+	-	2
25. Teotônio et al 2020	+	+	+	+	-	4

Key: 1. Sample representativeness (=65% of the sample), 2.sample size> 600 participants, 3. response rate>80%, 4. study used validate measurement tools with appropriate cut-offs and 5 control of confounding variables or used multiple regression

Quality assessment

We used the Modified Newcastle-Ottawa quality assessment scale tool to assess the quality of included papers and only 3 papers scored five out five (5/5) (26, 32, 35). Ten papers scored four out of five (4/5) (7, 28, 29, 19, 31, 27, 41, 34, 20, 39). Eight studies scored three out five (3/5) (30, 40, 34, 21, 24, 25, 36). Only three papers (22, 37, 23) scored 2 out five making it the lowest score and therefore low quality.

Quality of Life and Factors Associated to the low HRQoL

EQ-5D

The EQ-5D is a preference and generic quality of life instrument to valuate and describe health related quality of life; the higher the index, the better the health. It describes health in term of five dimensions; mobility, self-care, usual activities, pain/discomfort, and anxiety/depression(42). A utility score can be generated from the five dimensions based on a published algorithm with a

value of 0 for death and 1 for perfect health. Eight studies (22,40, 19, 32, 24,25, 37) have used the EQ-5D to assess the quality of life for the general population during COVID-19 pandemic. The mean score using EQ-5D estimated at 0.89 [95% CI 0.66-1.13]. By using EQ-5D, authors(22) reported the results (before and during confinement) that the quality of life was affected in the five health dimensions; mobility 87%(87%), self-care 97%(93%), usual activities 82%(89%), pain/discomfort 70%(78%) and anxiety/depression 44%(66%). His comparison on the two samples showed that during confinement, peoples had lower scores of HRQoL at 0.86 (p<0.001) as compared to before confinement whose score was 0.91(22). Female gender was affected with lower scores of HRQoL than their counterpart male on both utility (0.85; P=<0.0001 and VAS (78.49; P=0.004) and (utility =0.89 and VAS =83.78) respectively. Marital status was significantly associated to EQ-5D utility (P=0.002) and VAS (P=0.005) scores, widowed had the worst HRQoL (utility=0.43 and VAS=48.75) compared to single (utility=0.87 and VAS=80.09), married (utility=0.86 and VAS=81.43), and separated (utility=0.89 and VAS=80.15) participants. Participants with university education had the higher EQ-5D utility score (0.88; p<0.001) and age did not have a significant impact. A study done in Belgium and Netherlands also evaluated the quality of life using EQ5D as well, a minority in both countries felt stressed with 27% and 14% respectively(40). The majority reported concerns about their personal current and future financial situation (59 and 48% respectively) and the national economies (88 and 86%). Specifically, in Belgium, the EQ-5D before COVID-19 measured 0.82 (95% CI; 0.80–0.84) and during COVID-19 measures 0.79 (95% CI; 0.77–0.81). In Netherlands, before COVID, 0.85 (95% CI; 0.83–87) and during COVID-19 outbreak, it was 0.84 (95% CI; 0.82–0.86). Chen et al. (2021), using EQ5D concluded that the mean EQ-5D score and VAS were 0.99 and 93.5. Their multiple linear regression showed that the quality of life measure was related to physical activities ($\beta = 0.006$) and

keeping home ventilation (β = 0.063) in Daqing, and were related to wearing a mask when going out (β = 0.014), keeping home ventilation (β = 0.061), other marital status (β = − 0.011), worry about the epidemic (β = − 0.005) and having a centralized or home quarantine (β = − 0.005) in Taizhou (19). Using EQ5D**,** authors concluded that those quarantined at home experienced higher levels of anxiety and a lower HRQoL compared with the pre-COVID-19 pandemic population. Females and elderly individuals experienced the highest levels of anxiety and poorest HRQoL (OR not reported) (32). Other authors(20) using the same instrument EQ5D reported that the risk of pain/discomfort and anxiety/depression in general population in China raised significantly with aging, with chronic disease, lower income, epidemic effects, worried about get COVID-19 during the COVID-19 pandemic (OR not reported) (20). Tran et al. (2020) With the same instrument EQ5D (n=341) reported that 66.9% of household income loss was due to the impact of COVID-19. The mean score of EQ-5D and EQ-VAS was 0.95 (SD ± 0.07) and 88.2 (SD ± 11.0) respectively. The domain of Anxiety/Depression had the highest proportion of reporting any problems among 5 dimensions of EQ-5D (38.7%). Being female, having chronic conditions and living in the family with 3–5 members were associated with lower HRQOL score (OR not reported) (24). Vu et al. (2020) using EQ5D reported the highest mean EQ-VAS at 90.5 (SD: 7.98) among people in government quarantine facilities, followed by 88.54 (SD: 12.24) among general population and 86.54 (SD 13.69) among people in self-isolation group(25). The EQ-5D value was reported the highest among general population at 0.95 (SD: 0.07), followed by 0.94 (SD: 0.12) among people in government quarantine facilities, and 0.93 (SD: 0.13) among people who put themselves in self-isolation. Overall, most people, at any level, reported having problems with anxiety and/or depression in all groups.

WHOQoL-BREF

The WHO Quality of Life BREF (WHOQoL-BREF) is a 26-item instrument with four domains: physical health (7 items), psychological health (6 items), social relationships (3 items), and environmental health (8 items); Each item of the WHOQoL-BREF is scored from 1 to 5 on a response scale, which is stipulated as a five-point ordinal scale. The scores are then transformed linearly to a 0–100-scale. The physical health domain includes items on mobility, daily activities, functional capacity, energy, pain, and sleep whereas the psychological domain includes self-image, negative thoughts, positive attitudes, self-esteem, mentality, learning ability, memory concentration, religion, and the mental status. The social relationships domain contains questions on personal relationships, social support, and sex life and the environmental health domain covers issues related to financial resources, safety, health and social services, living physical environment, opportunities to acquire new skills and knowledge, recreation, general environment and transportation. Domain scores are graded in a positive direction and the higher scores denote higher quality of life(43). The average of the WHOQoL scores was estimated at 50.55% 95% CI [32.19-68.90]. Authors by using WHOQoL-BREF reported a quality of life affected with a score of 39% (CI not reported) and according to authors, males were more affected with OR = 1.96 (95% CI = 1.31–2.94); participants aged 26 to 35 years OR = 5.1; (95% CI = 1.33–19.37); non-Saudi participants OR = 1.69 (95% CI = 1.06–2.57); individuals with chronic diseases OR = 2.15 (95% CI = 1.33–3.48); those who lost their job OR = 2.18 (95% CI = 1.04–4.57) and those with depression OR = 5.70 (95% CI = 3.59–9.05), anxiety OR = 5.47; (95% CI = 3.38–8.84), and stress OR = 6.55 (95% CI = 4.01–10.70) (7). In 2021, a study (28) concluded that higher psychological QoL reduced the odds of depressive symptoms OR = 0.83 (95% CI = 0.69–0.99, p = 0.032) and depressive with comorbid anxiety symptoms OR = 0.82, (95% CI = 0.68–0.98, p = 0.041), whereas higher physical health QoL OR = 0.85, (95% CI = 0.75–0.97, p = 0.021) and social relationship

QoL OR = 0.70, (95% CI = 0.55–0.90, p = 0.009) reduced the odds of anxiety symptoms(28). Al-In 2020, a study(29) had reported a mean for total QoL score of 73.21 (SD ¼ 16.17). The mean general QoL and health scores were 3.15 (SD ¼ 0.94) and 3.40 (SD ¼ 0.95). As for the four QoL subscales, the mean scores in each domain were as follows: 18.04 (SD ¼ 4.39) for physical health, 17.65 (SD ¼ 3.77) for psychological health, 8.69 (SD ¼ 2.67) for social relationships, and 22.29 (SD ¼ 5.84) for environment(29). Choi et al (2021), using the same QoL scale reported that 69.6% of participants were worried about contracting COVID-19, and 41.4% frequently suspected themselves of being infected whereas 29.0% were concerned by the lack of disinfectants. All of these findings were associated with poorer HRQoL in the physical and psychological health, social relationships, and environment domains (OR not reported). 47.4% of participants were concerned that they may lose their job because of the pandemic and 39.4% were bothered by the insufficient supply of surgical masks(31). The results of a study(27) showed statistically significant difference in QoL depending on a number of variables, including sex, area of residence in Italy, and being diagnosed with a medical/psychiatric condition (OR not reported). The overall average score at the WHOQoL-BREF was 54.48 (SD = 7.77). The item with the lowest scores was 14 (about the use of spare time), given that 932 (41.4%) participants reported to have little or no time for leisure at the time of data collection. Regarding the other three domains of the WHOQoL, items with lowest scores were: item 15 for the physical domain, as 1019 (45.3%) participants reported little or no possibility to do physical activity; item 5 for the psychological domain, with 712 (31.6%) respondents reporting that they were not enjoying their lives at the time of data collection, and item 21 for social relationships, as 843 (37.4%) respondents reported that they were little or not at all satisfied with their sexual life(27). A research in 2021(33) reported that COVID-19 has had a wide impact on the general population, with the potential for negative secondary impacts. Women,

young adults, and the unemployed are at high risk for secondary effects (ORs not available). Another study (35) scores on the social relationships QoL domain were lower among participants who had a family member or friend with COVID-19 and among those who engaged in negative forms of spiritual religious coping (SRC). The quarantine during the COVID-19 pandemic has limited personal contact with family and friends, adversely affected sexual activity, and has restricted other activities that are assessed in the social relationships QoL domain. Positive forms of spiritual religious coping (SRC) were associated with better scores on this domain, as reported in other studies(33). In 2020, a study (36) highlighted that approximately one in three individual experienced mild-to-severe depressions during the nationwide movement control order (MCO). The varied impact of the pandemic on mental health could be due to different population characteristics and coping strategies used. Identifying those at higher risk to develop depression during movement control order (MCO) for COVID-19 pandemic could help mental healthcare service providers to plan services for those susceptible, thereby mitigating the pandemic's effect on quality of life(36). The results of a study (38) reported that most would expect quality of life to be challenged during a global pandemic; however, when behavioral health assessed as a component of overall quality of life, longer term outcomes became concerning. Both past and current mental health were strong predictors of quality of life. Participants who felt socially isolated reported personal health effects, or COVID-19 suspected or diagnosed participants reported lower quality of life (scores not reported) (38).

SF12/SF-8/ SF36

Short form (SF12/SF8/SF36)(34, 23, 21) are generic health survey short-forms (don't use preference based approach) to assess quality of life which are used in research and clinical practice, health policy and general surveys. It assesses eight health concepts (score is 0 to 100, 0 equivalent

to maximum disability health and 100 is equivalent to no disability); physical functioning, role physical, bodily pain, general health, vitality, social functioning, role emotional and mental health. SF12 and SF 36 can be used interchangeably according to Wee *et* al.(2008). The SF-8 is a short version of the original 36-item Short Form Health Survey (SF-36) with the same eight domains and is a generic multipurpose short-form quality of life instrument developed by the RAND Corporation and the Medical Outcomes Study (MOS) in the 1980s, 18 with two PCS and mental component summary (MCS) dimensions. Samlani *et* al. (2020) by using SF 12/8 (Chinese) scale, all participants obtained a total average score of 70.60 (±13.1) with a mental health score (MCS) of 34.49 (±6.44) and a physical health score (PCS) of 36.10 (± 5.82). The physical (PCS) and mental (MCS) scores of participants with chronic diseases were 32.51 (±7.14) and 29.28 (±1.23), respectively. Overall, the participants' PCS and MCS scores suffered from chronic diseases and the elderly participants were lower than those of young participants without comorbidities(23). López *et* al (2021) reported the following results using SF-36; the presence of pain in subjects undergoing confinement is persistent, with varying intensity and frequency based on age, gender, physical activity, and work status (ORs not reported). In any of these conditions, the quality of life of the subjects in confinement has been severely affected(34). Qi *et* al. 2020 using –SF8 (Chinese), participants' average physical component summary score (PCS) and mental component summary score (MCS) for HRQoL were 75.3 (SD = 16.6) and 66.6 (SD = 19.3), respectively. More than half of participants (53.0%) reported moderate levels of stress. Significant correlations between physical activity participation, HRQoL, and levels of perceived stress were observed ($p < 0.05$). Prolonged sitting time was also found to have a negative effect on HRQoL ($p < 0.05$) (21)

EQLS

European Quality of Life Survey (EQLS) is a 2012 EQLS version which considers the following dimensions; employment and work-life balance, family and social life, health and public services, home and local environment, quality of society, social exclusion and community involvement, standard of living and deprivation, subjective well-being which is designed for the general population. Campbell and Davison (2021) by using EQLS found that there are strong relationships between HRQoL and income, disability and living arrangement as well as social isolation and Disability and living arrangement(37). Correlation and multiple regression analyses showed a strong relationship between social isolation, gratitude, uncertainty and HRQoL, with social isolated being a significant predictor (OR not reported).

GH12

The General Health Questionnaire (GHQ) is a measure of current mental health and since its development by Goldberg in the 1970s it has been extensively used in different settings and different cultures. The questionnaire was originally developed as a 60-item instrument but at present a range of shortened versions of the questionnaire including the GHQ-30, the GHQ-28, the GHQ-20, and the GHQ-12 is available. GH12 is a 12-item validated questionnaire assessing the quality of life, it provides a reliable, valid, and brief assessment of QoL(48). The 12-item self-rated questionnaire measures physical health, psychological health, social relationships, and environment during the past 2 weeks. Each item is rated on a 4-point Likert scale (“less than usual”, “no more than usual”, “rather more than usual”, “much more than usual”), referring to the past 2 weeks, with a range of total scores from 0 to 36. Higher scores indicate a more problematic situation and there are different cut-offs to consider: Scores ≥ 15 are “problematic” and scores ≥ 19 as “very problematic”. Bonichini & Tremolada (2021) reported that the mean GH12 score in participants amounted to 17.86 (SD= 5.85), reflecting a contingent moderate stressful impact on

QoL. GH12 identified 39% of respondents as having subclinical QoL scores (score ≥ 15). 24.5% of such respondents as having very problematic scores (score ≥ 19), and 36.5% of such respondents as having normal scores (score < 15). Analysis of variance (ANOVA) showed there was a significant difference ($F(2, 1.836) = 5.50$, $p = 0.004$, $\eta^2 = 0.01$) in mean GH12 scores(26)

COVID-19 QoL questionnaire

The COV19-QoL is a 6-item scale covering main areas of quality of life in relation to mental health. The first item covers patients' feelings about the impact of the current pandemic on their quality of life in general. The second and third ones include participants' perceptions of possible mental and physical health deterioration. The latter one relates to anticipatory anxiety regarding physical health experienced due to perceiving different levels of risk of being contaminated. The virus represents direct risk to physical health of a person. Although most people will not be infected, it can be expected that the majority are concerned with the risk of being infected. The fourth and fifth items measure the levels of anxiety and depression due to the pandemic, respectively(41) . The last item includes the extent to which patients perceive their personal safety is now in danger. All items included a 5-point Likert scale (1 – "totally disagree" to 5 – "completely agree") and assess a period of last 7 days. Total scores are calculated by averaging the scores on all the items. A higher score indicates greater perceived impact of the pandemic on one's quality of life(49). The results of Khodami et al. (2020) showed that Quality of life is significantly decreased over time, perceived stress level raised significantly and an increased level of difficulty in emotion regulation has happened. Younger peoples and individuals who had a worsening quality of life response tended to show more stress and emotion regulation problems(41). Mohsen et al. (2022) using COVID-19 on Quality of life scale, the total COV19-QoL scale score was 2.3±0.6. Two items show the highest mean with 2.6±0.7 (quality of life in general and perception of danger

on their personal safety) indicating the poorest quality of life regarding these 2 items. However, the lowest mean score was related to the perception of mental health deterioration (1.9±0.8). Significant variables in the bivariable analysis revealed that sex (regression coefficient=0.1 (95% CI(0.02 to 0.2), p value=0.02), monthly income (regression coefficient (95% CI)=0.1 (0.004 to 0.2), p value=0.04), knowing someone infected with COVID19 (regression coefficient (95% CI)=0.15 (0.08 to 0.3), p value=0.001), and data collection time (regression coefficient (95% CI)=0.1 (0.006 to 0.2), p value=0.04) were the independent predictors for overall QoL scale score(39)

MLT

The 9- item My Life Today (MLT) scale was used to measure the participants' perceptions of various life domains, including the assessment of life in general. The participants responded to the question, “What is your life like at the present?”, and rated each item on a 6- point scale (1 = not good at all to 6 = very good). An overall index of quality of life was calculated by summing up the scores in all items. Recent studies utilized MLT as a valid instrument in assessing people's life quality with an internal consistency of $\alpha = 0.82$. Aruta et al. (2021) by using MLT questionnaire, the results of the path analysis indicated a good data- model fit: ($\chi^2 = 4.97$, df = 2, p = 0.08; CFI = 0.99, TLI = 0.96, SRMR = 0.02, RMSEA [90% CI] = 0.06 [0.000 − 0.13]). The direct effects of safety at home (B = −0.27, $\beta = -0.21$, SE = 0.05, $p \leq 0.001$), TPIs (B = −0.19, $\beta = -0.27$, SE = 0.05, $p \leq 0.001$), and financial difficulties (B = 0.15, $\beta = 0.18$, SE = 0.05, $p \leq 0.001$) on psychological distress were found to be significant**.** Direct effects of safety at home (B = 0.19, β = 0.22, SE = 0.05, $p \leq 0.001$), TPIs (B = 0.18, $\beta = 0.27$, SE = 0.04, $p \leq 0.001$), financial difficulties (B = −0.15, $\beta = -0.21$, SE = 0.05, $p \leq 0.001$), and psychological distress (B = −0.29, $\beta = -0.34$, SE = 0.04, $p \leq 0.001$) on quality of life were found to be significant**.** Results indicated that

psychological distress partially mediated the positive influence of safety at home (B = 0.06, β = 0.07, SE = 0.02, $p \leq 0.001$) and TPIs (B = 0.06, β = 0.09, SE = 0.02, $p \leq 0.001$) on quality of life (30). These findings indicate that psychological distress is a psychological mechanism that can partly explain why socio- ecological factors (i.e., safety at home, financial difficulties, and trust in institutions) impact the quality of life of Filipino adults during the COVID- 19.

Discussion

Findings of included studies demonstrated how COVID-19 pandemic reduced the quality of life of the general population worldwide. Different factors influenced directly or indirectly the change of quality of life. Researchers utilized different quality of life measurement scales among them EQ-5D leading the pool of measurement scales followed by WHOQoL-BREF then SF12/SF-8/ SF36 as 3rd scale as well as others. For studies which used EQ-5D to assess the impact of quality of life, all five dimensions (mobility, self-care, usual activities, pain/discomfort and anxiety/depression) were affected significantly with a mean EQ-5D score of 0.92 (below the EQ5D score before COVID-19) with 95% CI [-1.865-2.048] with the lowest score of 0.79 at 95% CI (not reported) and upper score of 0.99 at 95% CI (not reported) (22). The mean WHOQoL-BREF score was estimated at 50.55 with a 95% CI [32.19, 68.90]. Other instruments such as SF12 scored 70.60 with 95% CI [57.5, 83.7], SF8 scored QoL at 75.3 with 95% CI [58.7, 91.9] and SF36 (score not reported). In low and middle income countries (LMICs) such as Morocco(22) using EQ-5D reported low HRQoL during confinement as compared to before in the 5 health dimensions; mobility 87%(87%), self-care 97%(93%), usual activities 82%(89%), pain/discomfort 70%(78%) and anxiety/depression 44%(66%) with HRQoL at 0.91.5 ($p<0.001$) before and 0.86 (0.001) after confinement. Whereas in high income countries (HICs), Belgium for example using EQ-5D before

COVID-19 HRQoL measured 0.82 (95% CI; 0.80–0.84) and during COVID-19 measures 0.79 (95% CI; 0.77–0.81), the same with Netherlands, before COVID-19 EQ-5D measured 0.85 (95% CI; 0.83–87) before and during COVID-19 0.84 (95% CI; 0.82–0.86). In China, Chen et al. (2021), using EQ5D concluded that the mean EQ-5D score and VAS were 0.99 before COVID-19 and 93.5 during COVID-19. When compared HICs and LMICs, both countries were affected significantly by COVID-19 and this was exacerbated by confinement. These results are in line with those of Ping et *al*.(2020) in China, with an average score EQ-5D of 0.949 and VAS score 85.52(19). Nine published papers assessed HRQoL using WHOQoL(7, 28, 29, 31, 27, 33, 35, 36, 38) and their mean score was 50.55% with 95% CI [32.19-68.90]. The lower the score, the lower the quality of life. On the other hand, using EQ-5D, the mean score was estimated at 0.89 with 95% CI [0.66-1.13] with the same trend, the lower the score, the lower the quality of life. Different factors that contributed to low quality of life have been identified among them; age, gender, education level, marital status, financial constraints, confinement, fear of being contaminated and individual with other chronic conditions. The two measurement scales were the most utilized instruments as compared to the other scales and their results show a considerable reduced quality of life. Using WHOQoL-BREF (7) reported a quality of life affected with a score of 39% (CI not reported) and according to authors, males were more affected probably because in developing countries, males are responsible of financial support to the family and because of that, they may fear either confinement which affects job market or else being contaminated and not able to work for their families. Concerning age, participants aged 26-35years were more affected and the reason may be because most peoples of this age bracket are the young couples or single mothers therefore the young fathers were worried about their families and finances if they are quarantined. Females were more affected than their counterparts according to (22) this may be due to the fact that females

naturally are the nuclear parts of a family and their emotions towards the family therefore become much worried than males. Widowers had the worst quality of life and this may be due to their worries about their life and that of their children with less psychological support from their spouse. Individuals with chronic diseases (hypertension, Type2 diabetes, asthma, stress, anxiety, depression, etc…) had a high risk of low quality of life and this might be because they are vulnerable to COVID-19 with high fear of contamination therefore pushing them to low quality of life. Other factors such as confinement, financial constraints, fear of being contaminated with COVID-19 and having a contaminated family member increased the likelihood of anxiety, stress and depression therefore leading to the low individual quality of life. The main reason of stress due to confinement is due to financial constraint because a confined person is not allowed to work and generate income to sustain the family during the pandemic.

Strengths and Weaknesses

First and the foremost, the strength of this review is that, it was conducted according to the international guidelines for systematic reviews after registration of the protocol in international database PROSPERO. Secondly, it was conducted two and half years after the pandemic begun and authors already have published enough papers to allow robust systematic synthesis of results. And the results can be generalized as papers were searched Worldwide with a sample size of 22,967 participants.

There were also some limitations; the fact that included papers assessed the quality of life using different tools, this makes the comparison among studies very difficult. We also searched papers in English only leaving probably out some studies. The fact that we searched only 3 databases and

a search engine, relevant articles might have been missed. The generalizability should be done with caution.

Conclusion

This systematic review confirms that the COVID-19 pandemic affected negatively health related quality of life of the general population worldwide. Several factors have been identified as influencing factors of COVID-19 pandemic in regards to quality of life such as age, sex, marital status, education, peoples living with chronic diseases, confinement and financial constraints among others, etc…. There was a difference between the impact of COVID-19 in general population in high income countries and low and middle income countries. Three main quality of life scales were used to assess the quality of life of the general population among them; WHO-QoL-BREEF, EQ-5D, SF and others. The findings of this review will be useful for policy makers, public health managers and researchers to facilitate the management plan of quality of life of the general population and in the prevention during future pandemics. There is a need for more studies evaluating the impact of COVID-19 on general population using other approved scales for a robust comparison.

Acknowledgements

The authors acknowledge the moral support from their families, friends and colleagues

References

1. WHO. COVID 19 Public Health Emergency of International Concern (PHEIC) Global Research and Innovation Forum: Towards a Research Roadmap. Glob Res Collab Infect Dis Prep [Internet]. 2020;1–10. Available from: https://www.who.int/publications/m/item/covid-19-public-health-emergency-of-international-concern-(pheic)-global-research-and-innovation-forum

2. Nam NH, Tien PTM, Van Truong L, El-Ramly TA, Anh PG, Hien NT, et al. Early centralized isolation strategy for all confirmed cases of COVID-19 remains a core intervention to disrupt the pandemic spreading significantly. PLoS One [Internet]. 2021;16(7 July):1–17. Available from:

http://dx.doi.org/10.1371/journal.pone.0254012

3. Jiaqi Xionga, Orly Lipsitzc, Flora Nasric, Leanna M.W. Luic, Hartej Gillc LP, David Chen-Lic, Michelle Iacobuccic, Roger Hoe, f, Amna Majeedc, Roger S. McIntyrea, b, c D. Impact of COVID-19 pandemic on mental health in the general population: A systematic review. J Affect Disord. 2020;(January).

4. Aronson KI, Suzuki A. Health Related Quality of Life in Interstitial Lung Disease: Can We Use the Same Concepts Around the World? Front Med. 2021;8(October):1–12.

5. Taylor S, Paluszek MM, Rachor GS, Mckay D, Gordon J, Asmundson G. Substance use and abuse, COVID-19-related distress, and disregard for social distancing: A network analysis. Addict Behav. 2020;(January).

6. Nobari H, Fashi M, Eskandari A, Villafaina S, Murillo-Garcia Á, Pérez-Gómez J, et al. Effect of COVID-19 on Health-Related Quality of Life in Adolescents and Children: A Systematic Review. Int J Environ Res Public Heal [Internet]. 2021;18:4563. Available from: https://doi.org/10.3390/ijerph

7. Algahtani FD, Hassan SUN, Alsaif B, Zrieq R. Assessment of the quality of life during covid-19 pandemic: A cross-sectional survey from the kingdom of Saudi Arabia. Int J Environ Res Salud Pública. Int J Environ Res Public Health [Internet]. 2021;18(3):1–12. Available from: https://www.ncbi.nlm.nih.gov/pmc/articles/PMC7863741/pdf/ijerph-18-00847.pdf

8. Lindqvist Bagge AS, Carlander A, Fahlke C, Olofsson Bagge R. Health-related quality of life (FACT-GP) in Sweden. Health Qual Life Outcomes. 2020;18(1):1–10.

9. Matos RA da C, Akutsu R de CC de A, Zandonadi RP, Botelho RBA. Quality of life prior and in the course of the covid-19 pandemic: A nationwide cross-sectional study with brazilian dietitians. Int J Environ Res Public Health. 2021;18(5):1–13.

10. Moher D, Liberati A, Tetzlaff J, Altman DG, Altman D, Antes G, et al. Preferred reporting items for systematic reviews and meta-analyses: The PRISMA statement. PLoS Med. 2009;6(7).

11. Viechtbauer W. Bias and efficiency of meta-analytic variance estimators in the random-effects model. J Educ Behav Stat. 2005;30(3):261–93.

12. van Aert RCM, van Assen MALM, Viechtbauer W. Statistical properties of methods based on the Q-statistic for constructing a confidence interval for the between-study variance in meta-analysis. Res Synth Methods. 2019;10(2):225–39.

13. Khan MS, Li L, Yasmin F, Khan SU, Bajaj NS, Pandey A, et al. Assessment of Heterogeneity in Heart Failure-Related Meta-Analyses. Circ Hear Fail. 2020;13(11):E007070.

14. Riley RD, Higgins JPT, Deeks JJ. Interpretation of random effects meta-analyses. Bmj. 2011;342(7804):964–7.

15. Viechtbauer W, Cheung MW-L. Outlier and influence diagnostics for meta-analysis. Res Synth Methods. 2010;1(2):112–25.

16. Gjerdevik M, Heuch I. Improving the error rates of the Begg and Mazumdar test for publication bias in fixed effects meta-analysis. BMC Med Res Methodol. 2014;14(1):1–16.

17. McShane BB, Böckenholt U, Hansen KT. Adjusting for Publication Bias in Meta-Analysis: An Evaluation of Selection Methods and Some Cautionary Notes. Perspect Psychol Sci. 2016;11(5):730–49.

18. Viechtbauer W. Conducting meta-analyses in R with the metafor. J Stat Softw. 2010;36(3):1–48.

19. Chen X, Xu Q, Lin H, Zhu J, Chen Y, Zhao Q, et al. Quality of life during the epidemic of COVID-19 and its associated factors among enterprise workers in East China. BMC Public Health. 2021;21(1):1–8.

20. Ping W, Zheng J, Niu X, Guo C, Zhang J, Yang H, et al. Evaluation of health-related quality of life using EQ-5D in China during the COVID-19 pandemic. PLoS One [Internet]. 2020;15(6):1–12. Available from: http://dx.doi.org/10.1371/journal.pone.0234850

21. Qi M, Li P, Moyle W, Weeks B, Jones C. Physical Activity, Health-Related Quality of Life, and Stress Among the Chinese Adult Population During the COVID-19 Pandemic. Int J Environ Res Public Health [Internet]. 2020;17(1):1–10. Available from: https://www.ncbi.nlm.nih.gov/pmc/articles/PMC7558071/pdf/ijerph-17-06494.pdf

22. Azizi A, Achak D, Aboudi K, Saad E, Nejjari C, Nouira Y, et al. Health-related quality of life and behavior-related lifestyle changes due to the COVID-19 home confinement: Dataset from a Moroccan sample. Data Br [Internet]. 2020;32:106239. Available from: https://doi.org/10.1016/j.dib.2020.106239

23. Samlani Z, Lemfadli Y, Ait Errami A, Oubaha S, Krati K. The impact of the COVID-19 pandemic on quality of life and well-being in Morocco. Arch Community Med Public Heal. 2020;11(2):130–4.

24. Tran BX, Nguyen HT, Le HT, Latkin CA, Pham HQ, Vu LG, et al. Impact of COVID-19 on Economic Well-Being and Quality of Life of the Vietnamese During the National Social Distancing. Front Psychol. 2020;11(September):1–9.

25. Vu MQ, Tran TTP, Hoang TA, Khuong LQ, van Hoang M. Health-related quality of life of the Vietnamese during the COVID-19 pandemic. PLoS One [Internet]. 2020;15(12 December):1–15. Available from: http://dx.doi.org/10.1371/journal.pone.0244170

26. Bonichini S, Tremolada M. Quality of life and symptoms of ptsd during the covid- 19 lockdown in Italy. Int J Environ Res Public Health. 2021;18(8).

27. Epifanio MS, Andrei F, Mancini G, Agostini F, Piombo MA, Spicuzza V, et al. The impact of COVID-19 pandemic and lockdown measures on quality of life among Italian general population. J Clin Med. 2021;10(2):1–19.

28. Leong Bin Abdullah MFI, Ahmad Yusof H, Mohd Shariff N, Hami R, Nisman NF, Law KS. Depression and anxiety in the Malaysian urban population and their association with demographic characteristics, quality of life, and the emergence of the COVID-19 pandemic. Curr Psychol. 2021;40(12):6259–70.

29. Al-Shannaq Y, Mohammad AA, Aldalaykeh M. Depression, coping skills, and quality of life among Jordanian adults during the initial outbreak of COVID-19 pandemic: cross sectional study. Heliyon [Internet]. 2021;7(4):e06873. Available from: https://doi.org/10.1016/j.heliyon.2021.e06873

30. Aruta JJBR, Callueng C, Antazo BG, Ballada CJA. The mediating role of psychological distress on the link between socio-ecological factors and quality of life of Filipino adults during COVID-19 crisis. J Community Psychol. 2022;50(2):712–26.

31. Choi EPH, Hui BPH, Wan EYF, Kwok JYY, Tam THL, Wu C. Covid-19 and health-related quality of life: A community-based online survey in Hong Kong. Int J Environ Res Public Health. 2021;18(6):1–12.

32. Ferreira LN, Pereira LN, da Fé Brás M, Ilchuk K. Quality of life under the COVID-19 quarantine. Qual Life Res [Internet]. 2021;30(5):1389–405. Available from: https://doi.org/10.1007/s11136-020-02724-x

33. Lipskaya-Velikovsky L. and Public Health Article COVID-19 Isolation in Healthy Population in Israel: Challenges in Daily Life. Int J Environ Res [Internet]. 2021;18:999. Available from: https://doi.org/10.3390/ijerph

34. Iglesias-López E, García-Isidoro S, Castellanos-Sánchez VO. COVID-19 pandemic: pain, quality of life and impact on public health in the confinement in Spain. Ann Palliat Med. 2021;10(4):4338–53.

35. Vitorino LM, Yoshinari Júnior GH, Gonzaga G, Dias IF, Pereira JPL, Ribeiro IMG, et al. Factors associated with mental health and quality of life during the COVID-19 pandemic in Brazil. BJPsych Open. 2021;7(3):1–8.

36. Yee A, Hodori N 'Aqilah M, Tung YZ, Ooi PL, Latif SABA, Isa HM, et al. Depression level and coping responses toward the movement control order and its impact on quality of life in the Malaysian community during the COVID-19 pandemic: a web-based cross-sectional study. Ann Gen Psychiatry [Internet]. 2021;20(1):1–9. Available from: https://doi.org/10.1186/s12991-021-00352-4

37. Campbell D, Davison J. The Impact of COVID-19 on the Health-Related Quality of Life of Individuals Living in Scottish Communities with High Infection Rates. Eur J Environ Public Heal. 2022;6(1):em0099.

38. Hansel TC, Saltzman LY, Melton PA, Clark TL, Bordnick PS. COVID-19 behavioral health and quality of life. Sci Rep [Internet]. 2022;12(1):1–10. Available from: https://doi.org/10.1038/s41598-022-05042-z

39. Mohsen S, El-Masry R, Ali OF, Abdel-Hady D. Quality of life during COVID-19 pandemic: a community-based study in Dakahlia governorate, Egypt. Glob Heal Res Policy [Internet]. 2022;7(1). Available from: https://doi.org/10.1186/s41256-022-00246-2

40. van Ballegooijen H, Goossens L, Bruin RH, Michels R, Krol M. Concerns, quality of life, access to care and productivity of the general population during the first 8 weeks of the coronavirus lockdown in Belgium and the Netherlands. BMC Health Serv Res. 2021;21(1):1–8.

41. M. Ahsan Khodami, M. Hassan Seif RSK et al. Perceived stress, emotion regulation and quality of life during the Covid-19 outbreak: A multi-cultural online survey. Ann Me´dico-Psychologiques. 2020;(January).

42. Obradovic M, Lal A, Liedgens H. Validity and responsiveness of EuroQol-5 dimension (EQ-5D) versus Short Form-6 dimension (SF-6D) questionnaire in chronic pain. Health Qual Life Outcomes. 2013;11(1):1–9.

43. Gil-Lacruz M, Cañete-Lairla M, Navarro J, Montaño-Espinoza R, Espinoza-Santander I, Osorio-Parraguez P. Validation of the WHOQOL-BREF Quality of Life Questionnaire in an Urban Sample of Older Adults in a Neighbourhood in Zaragoza (Spain). Healthcare. 2022;10(11):2272.

44. Huo T, Guo Y, Shenkman E, Muller K. Assessing the reliability of the short form 12 (SF-12) health survey in adults with mental health conditions: A report from the wellness incentive and navigation (WIN) study. Health Qual Life Outcomes. 2018;16(1):1–8.

45. Eurofound. European quality of life survey 2016: Quality of life, quality of public services, and quality of society. [Internet]. 2017. 76 p. Available from:

https://www.eurofound.europa.eu/publications/report/2017/fourth-european-quality-of-life-survey-overview-report

46. Montazeri A, Harirchi AM, Shariati M, Garmaroudi G, Ebadi M, Fateh A. Health and Quality of Life Outcomes | Full text | The 12-item General Health Questionnaire (GHQ-12): translation and validation study of the Iranian version. Health Qual Life Outcomes [Internet]. 2003;1(66):1–4. Available from: http://www.hqlo.com/content/1/1/66

47. Teotônio I, Hecht M, Castro LC, Gandolfi L, Pratesi R, Nakano EY, et al. Repercussion of COVID-19 pandemic on brazilians' quality of life: A nationwide cross-sectional study. Int J Environ Res Public Health. 2020;17(22):1–11.

48. Kim YJ, Cho MJ, Park S, Hong JP, Sohn JH, Bae JN, et al. The 12-item general health questionnaire as an effective mental health screening tool for general Korean adult population. Psychiatry Investig. 2013;10(4):352–8.

49. Shah R, Ali FM, Nixon SJ, Ingram JR, Salek SM, Finlay AY. Measuring the impact of COVID-19 on the quality of life of the survivors, partners and family members: A cross-sectional international online survey. BMJ Open. 2021;11(5):1–13.

50. Institute of Medicine. 2011. Finding What Works in Health Care: Standards for Systematic Reviews. Washington, DC: The National Academies Press. https://doi.org/10.17226/13059.

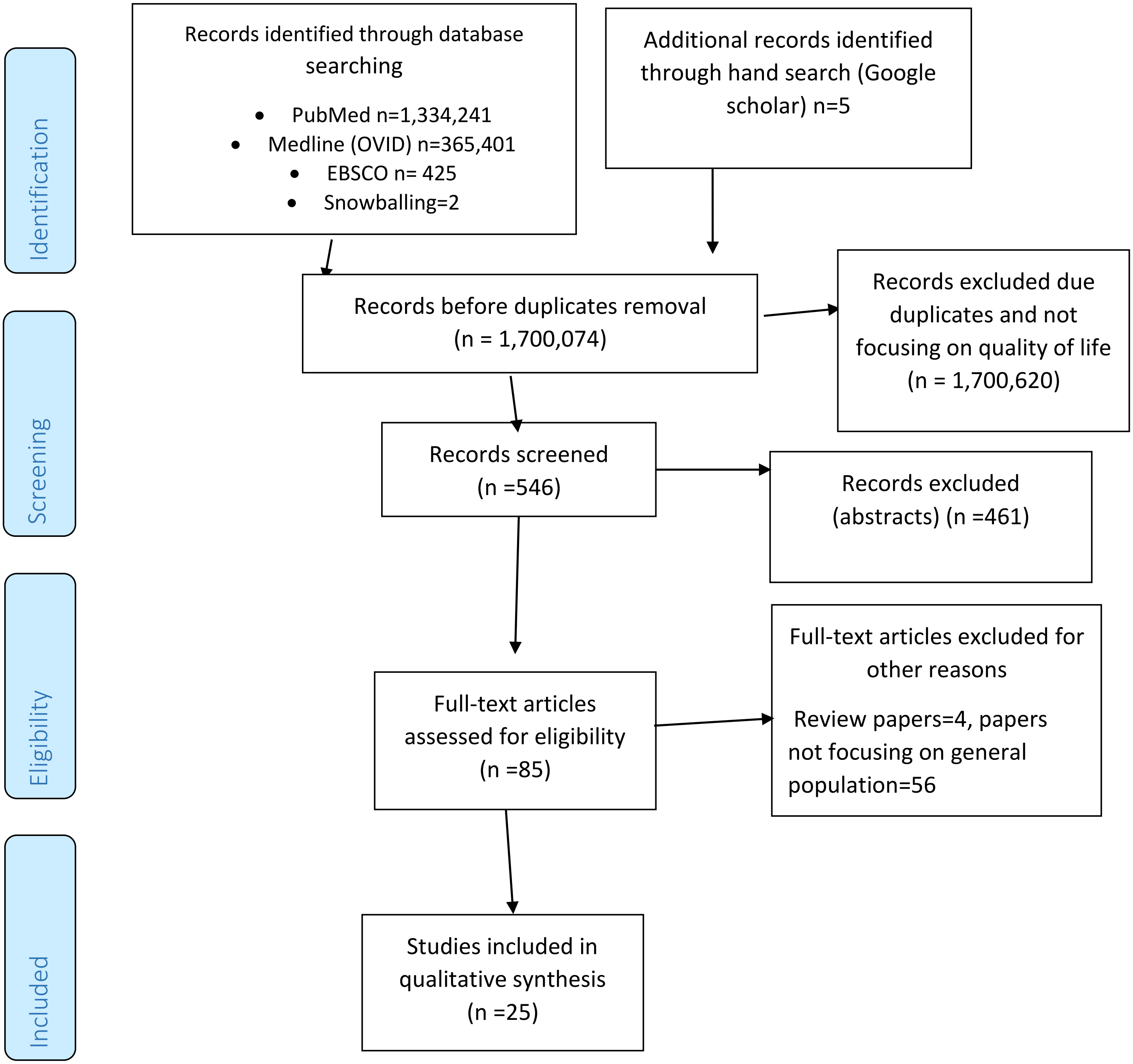

Fig1. Preferred Reporting Items for Systematic Reviews and Meta Analyses (PRISMA) flow diagram (10)

Figure2

Click here to access/download;Figure;Fig2 EQ5D TIFF forest.tiff

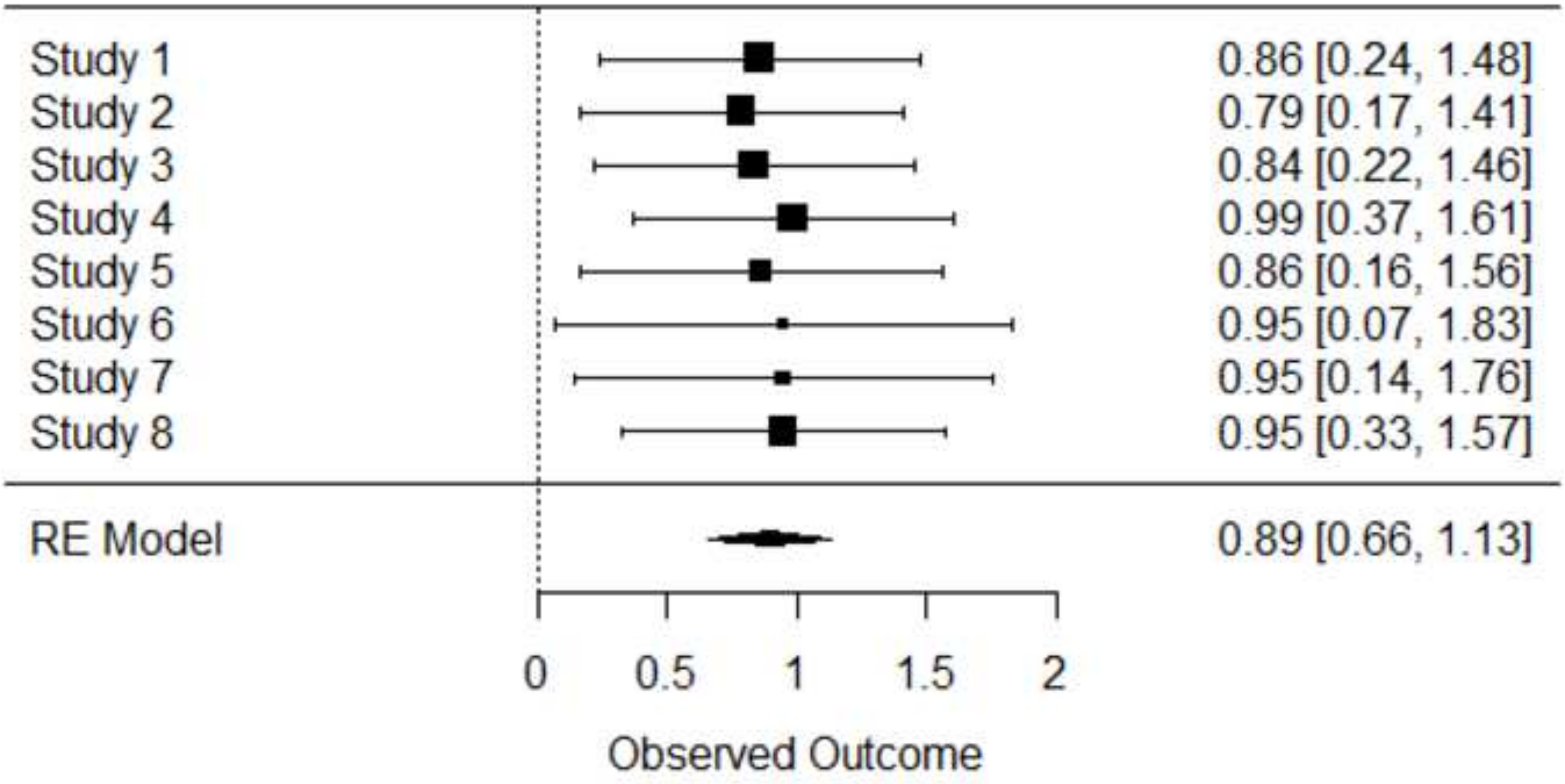

 Click here to access/download;Figure;Fig3 WHOQoL_TIFF_forest.tiff

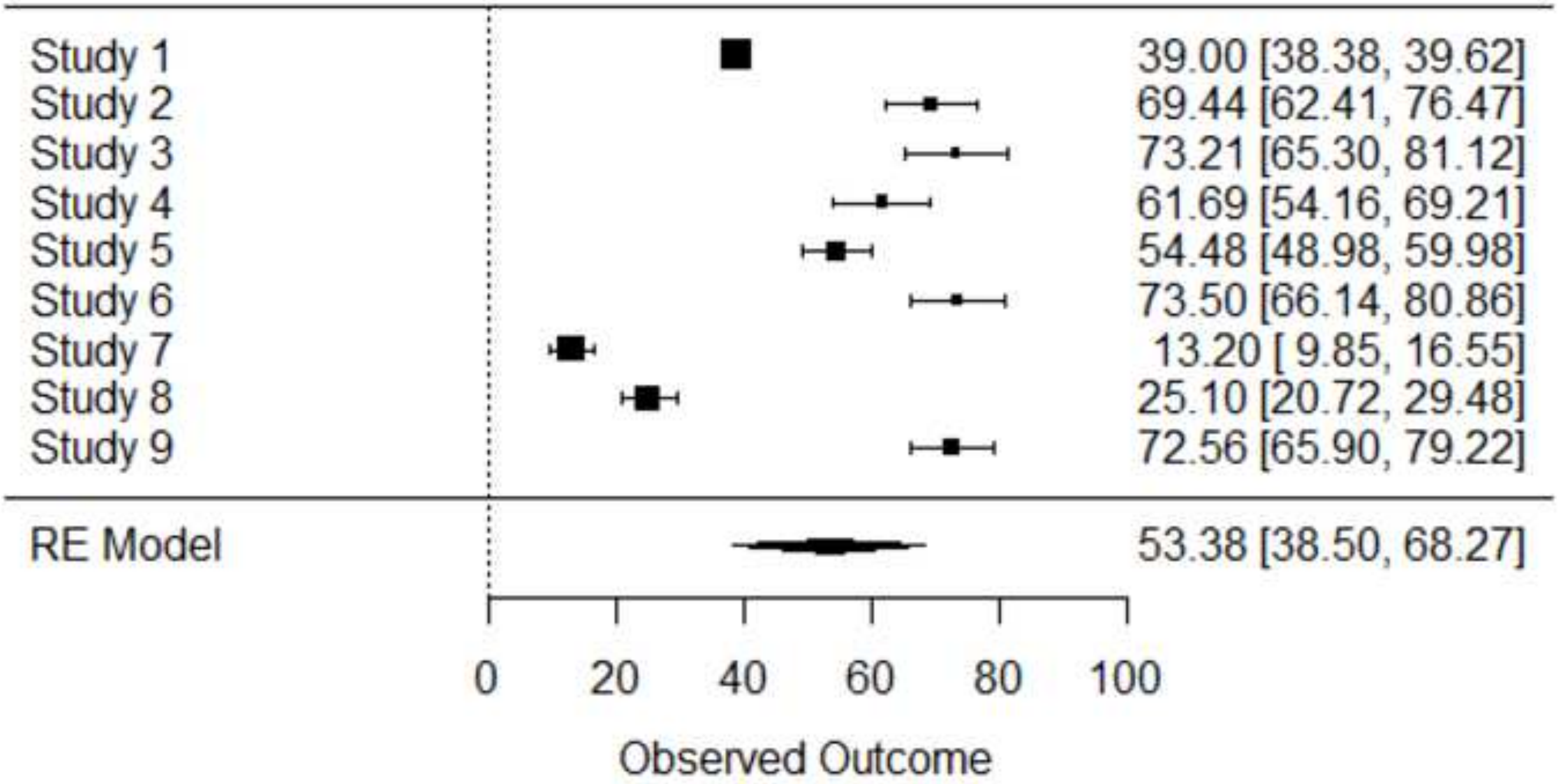

Figure4

Click here to access/download;Figure;Fig4 WHOQoL_TIFF_funnel.tiff

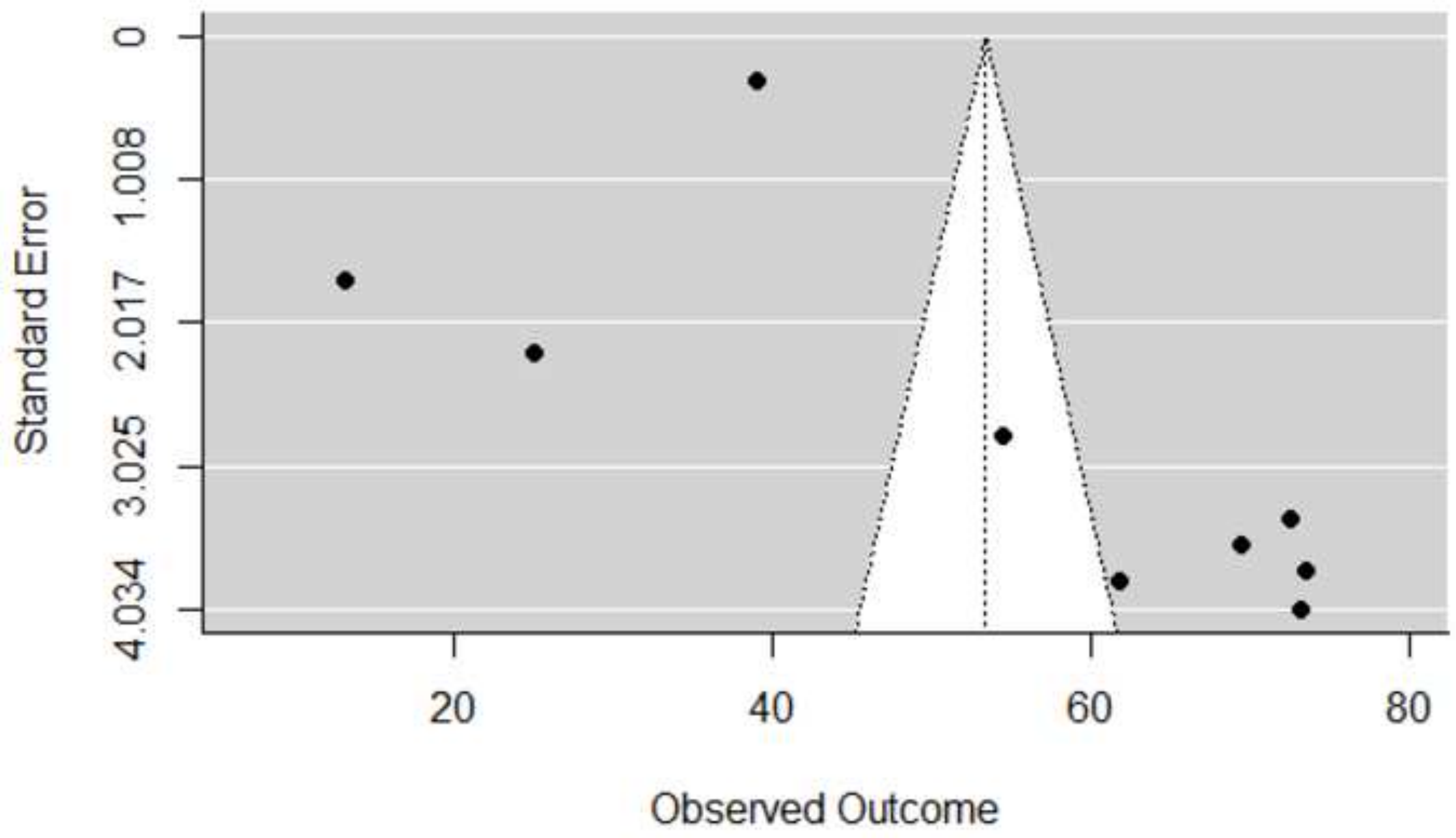

Figure5

Click here to access/download;Figure;Fig5 EQ5D TIFF funnel.tiff

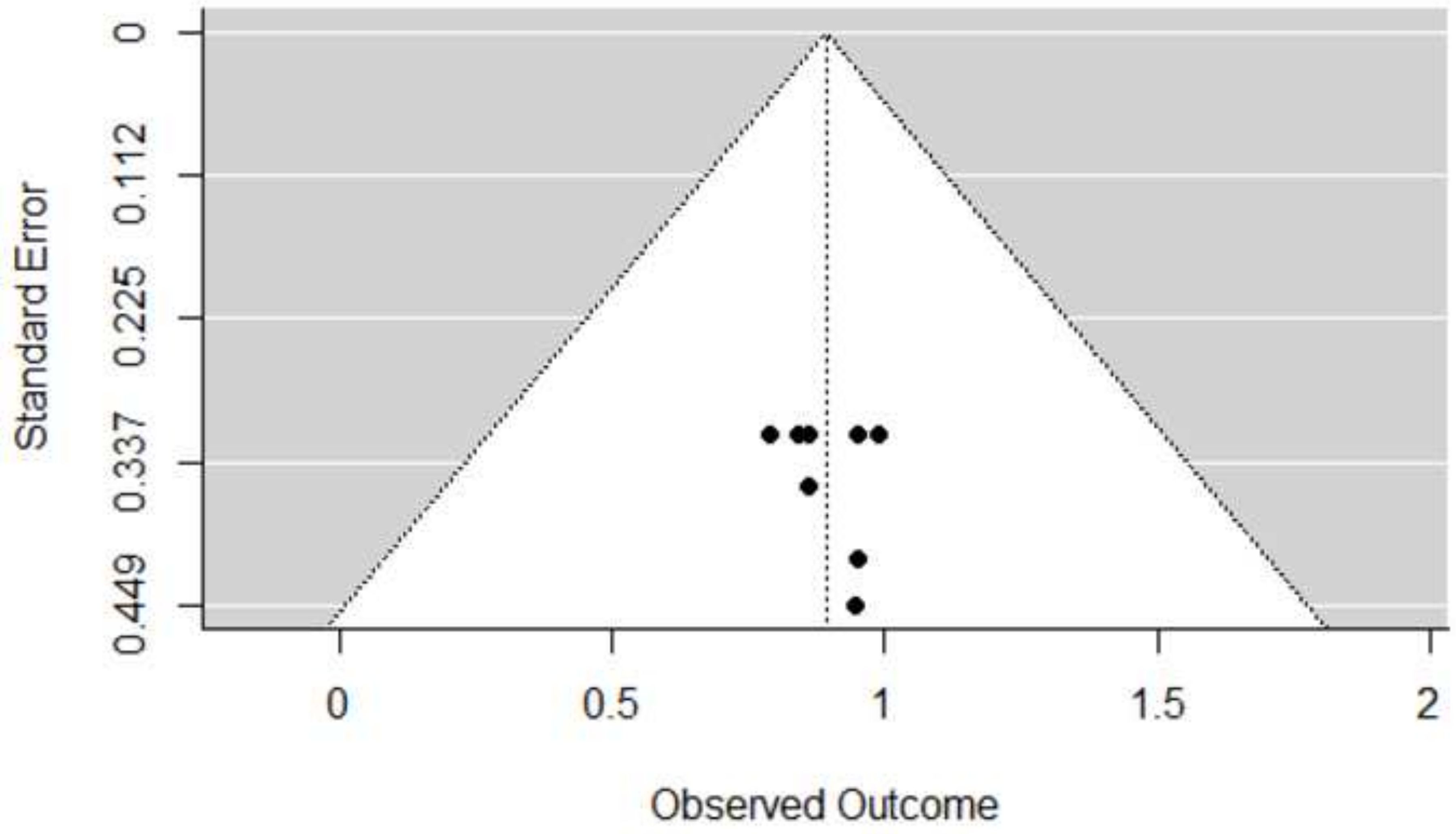

S1

Click here to access/download
Supporting Information
S1 HRQoL.docx

S2

Click here to access/download
Supporting Information
S2 PRISMA Checklist 2009 HRQoL.doc

S3

Click here to access/download

Supporting Information

S3 HRQoL.docx
