# Supplementary material for: Impact of COVID-19 on health-related quality of life in the general population: A systematic review and meta-analysis
Source: PLOS Glob Public Health. 2023 Oct 26;3(10):e0002137. doi: 10.1371/journal.pgph.0002137 (PMC10602258; doi:10.1371/journal.pgph.0002137)
Supplement: Attachment — Submitted filename: Response to Editor and reviewers.docx [file pgph.0002137.s005.docx]

Academic Editor

PLOS ONE Global Public Health

September 26^th^, 2023

Dear Academic Editor,

I hereby submit the response to reviewers for the manuscript entitled “Impact of COVID-19 on Health-Related Quality of Life in the General Population: a systematic review and meta-analysis”. The objective of this study is to assess the impact of COVID-19 and its related factors on the HRQoL in the general population. Here are the amended files; manuscript, manuscript with track changes, supplementary documents, cover letter showing corrections done and figures. We believe that we exhaustively done all required corrections. Here are the corrections effected;

**Academic editor**

1. Thank you for this important correction, we have amended the title accordingly now reading “Impact of COVID-19 on Health-Related Quality of Life in the General Population: a systematic review and meta-analysis

2. We have amended the figures to TIFF format. Kindly find attached

3. We have added a list of legends showing the figures and supplementary documents. Thank you

4. We considered English editing to improve the readability

5. We have reviewed the abstract and aim & objective have been added at the end of introduction

6. Methods: we have added the Inclusion and exclusion criteria under eligibility criteria however we have noticed that the information may be double because eligibility criteria contained inclusion and exclusion criteria. Kindly advise. Thank you

7. We have added a section on quality of life scores before and during COVID-19 as per your request. Thank you

8. We have explained the reason for including low quality papers in the manuscript.

9. We have revised the discussion section as per your advise we hope the arguments are stronger

**Review1**

1. we have compared the changes of quality of life due to some chronic conditions such as Type2 diabetes, Denge fever, HIV, frail eldery, injuries, skin disease and respiratory disease and changes of quality of life due to COVID-19. Thank you

2. We have added a section in the introduction on burnout, depression and anxiety in regards to COVID-19 and quality of life

3. We have added a paragraph explaining random-effect model

**Reviewer2**

1. The reduction of searched papers from the first search has been done (from 1,700,074 hits to 546) and explained accordingly

2. We have added the aim and objective of the research at the end of introduction

3. Information on the scale rangers has been added and standard values on healthy or unaffected populations have been given and the results have been explained in comparison with normal values

4. All information given twice has been deleted

We take this opportunity to thank you sincerely for your constructive feedback.

Your faithfully,

Desire Aime Nshimirimana
